# Herpes Simplex Virus Dances with Amyloid Precursor Protein while Exiting the Cell

**DOI:** 10.1371/journal.pone.0017966

**Published:** 2011-03-31

**Authors:** Shi-Bin Cheng, Paulette Ferland, Paul Webster, Elaine L. Bearer

**Affiliations:** 1 Department of Pathology and Laboratory Medicine, Alpert Medical School of Brown University, Providence, Rhode Island, United States of America; 2 House Ear Institute, Los Angeles, California, United States of America; 3 Departments of Pathology and of Neurosurgery, University of New Mexico School of Medicine, Albuquerque, New Mexico, United States of America; Hannover Medical School, Germany

## Abstract

Herpes simplex type 1 (HSV1) replicates in epithelial cells and secondarily enters local sensory neuronal processes, traveling retrograde to the neuronal nucleus to enter latency. Upon reawakening newly synthesized viral particles travel anterograde back to the epithelial cells of the lip, causing the recurrent cold sore. HSV1 co-purifies with amyloid precursor protein (APP), a cellular transmembrane glycoprotein and receptor for anterograde transport machinery that when proteolyzed produces A-beta, the major component of senile plaques. Here we focus on transport inside epithelial cells of newly synthesized virus during its transit to the cell surface. We hypothesize that HSV1 recruits cellular APP during transport. We explore this with quantitative immuno-fluorescence, immuno-gold electron-microscopy and live cell confocal imaging. After synchronous infection most nascent VP26-GFP-labeled viral particles in the cytoplasm co-localize with APP (72.8+/−6.7%) and travel together with APP inside living cells (81.1+/−28.9%). This interaction has functional consequences: HSV1 infection decreases the average velocity of APP particles (from 1.1+/−0.2 to 0.3+/−0.1 µm/s) and results in APP mal-distribution in infected cells, while interplay with APP-particles increases the frequency (from 10% to 81% motile) and velocity (from 0.3+/−0.1 to 0.4+/−0.1 µm/s) of VP26-GFP transport. In cells infected with HSV1 lacking the viral Fc receptor, gE, an envelope glycoprotein also involved in viral axonal transport, APP-capsid interactions are preserved while the distribution and dynamics of dual-label particles differ from wild-type by both immuno-fluorescence and live imaging. Knock-down of APP with siRNA eliminates APP staining, confirming specificity. Our results indicate that most intracellular HSV1 particles undergo frequent dynamic interplay with APP in a manner that facilitates viral transport and interferes with normal APP transport and distribution. Such dynamic interactions between APP and HSV1 suggest a mechanistic basis for the observed clinical relationship between HSV1 seropositivity and risk of Alzheimer's disease.

## Introduction

Herpes simplex virus type I (HSV1), an alpha herpesvirus, is endemic in the general population, causing life-long latent infections in neurons. Like many other viruses, after assembly in the nucleus HSV1 nucleocapsids transport outwards through the cytoplasm towards the cell surface both in epithelial cells and in neurons [1], [2], [3], [4], [5], [6], [7]. While anterograde transport of newly synthesized virus to the epithelial cell surface and from neuronal cell bodies to the mucosal membrane is crucial for viral propagation to a new host, neither the cellular nor viral molecular mediators are known.

How HSV1 coordinates assembly with transport remains an important unresolved question. Such coordinated assembly may differ between epithelial cells and neurons, and between different types of alpha herpesviruses, which has led to some controversy. Recent evidence suggests that the swine alpha herpesvirus, pseudorabies virus (PRV), travels inside membranated vesicles within neurons [8], a mechanism of anterograde transport also believed to be invoked in epithelial cells by HSV1 (reviewed in [9]). Newly synthesized HSV1 capsids travel outward from the nucleus either independently to be assembled with other components at the cell periphery [3], [6], [10], [11], together as enveloped particles inside a second, Golgi-derived, cellular membrane [12], [13], [14], [15], [16], [17], or both [1], [18], [19], [20]. Electron-microscopy of infected cells demonstrates capsids both free in the cytoplasm as well as inside intracellular membrane systems [9], [15], [16], [19].

To coordinate envelopment with transport, the virus must take advantage of cellular synthetic and transport machinery. Such co-option of transport machinery may underlie HSV1 cellular pathology, injuring cells by interfering with this normal important cellular process. Exploiting green-fluorescent-protein (GFP)-labeled HSV1 as a tool to uncover cargo motor receptors led us to discover that the cellular transmembrane glycoprotein, amyloid precursor protein (APP), is a component of isolated HSV1 intracellular viral particles, with ∼1,000 or more copies on average per particle [17]. Since altered APP is a known risk factor for Alzheimer's disease [21], [22], which has been linked to transport defects [23], [24], [25], [26], interactions of APP with HSV1 are potentially significant. Earlier, we found that isolated intracellular viral particles physically associated with APP are transported in the anterograde direction when injected into the giant axon of the squid [17]. Furthermore, a 15-amino acid “zipcode” from the cytoplasmic C-terminus of APP mediates anterograde transport of fluorescent beads in the axon [27]. Thus APP hitches exogenous cargo to cellular anterograde motor machinery for transport, which suggests one mechanism the virus may exploit to travel to the cell surface.

While APP is physically associated with viral particles isolated from infected cell cytoplasm, it was not among the proteins identified by mass spectroscopy of extracellular infectious particles [23]. Thus it is unknown whether APP joins viral particles during packaging inside the cell, or during the procedure to isolate viral particles, which includes disruption of infected cells, centrifugations and sucrose density fractionation, which could redistribute molecules not normally together.

We therefore set out to determine whether APP co-localizess with virus inside cells, and whether interplay between virus and APP-containing cellular membranes enhances transport of nascent virus from peri-nuclear region to the cell surface and/or affects APP transport dynamics. Two approaches were developed to assign viral particles as either out-going or in-coming: Synchronizing infection to a narrow time window and thus limiting in-coming viral particles during viral production; and live imaging. We used a GFP-tagged virus, VP26-GFP HSV1, to visualize viral particles as they form and travel. VP26-GFP labeling has proven to be a spectacular tool to image viral capsid movements [28]. VP26 is a small protein that attaches to the major capsid protein, VP5, by binding to the outer surface of hexons [29], [30]. VP26-GFP labels viral capsids without disturbing viral function [28], [31], [32]. Here we explored dynamic interactions between viral capsids and cellular APP inside epithelial cells during viral egress in synchronously infected cells. While epithelial cells and neurons may differ in details of transport mechanisms, the major mechanisms are likely to be the same: microtubule-based transport by kinesin and dynein families of molecular motors. For these studies we developed a method to limit infection to a short time window such that >90% of cytoplasmic capsids in productively infected cells represent newly synthesized virus. We then probed VP26-GFP-HSV1-infected cells for APP by quantitative immunohistochemistry and immunogold electron-microscopy, and also performed dual-color confocal time-lapse imaging of fluorescent-protein labeled capsids (VP26-GFP) interacting with labeled APP (APP-mRFP) inside living cells. We observed frequent dynamic interactions between cellular APP and out-going newly synthesized viral particles. APP interplay greatly increased the propensity of VP26-GFP capsids to move. These studies also reveal that HSV1 infection and subsequent capsid-APP interactions decrease the transport dynamics and provoke an abnormal subcellular distribution of cellular APP.

## Results

### Synchronizing infection to distinguish in-coming from out-going particles in the cytoplasm

**Figure 1:**
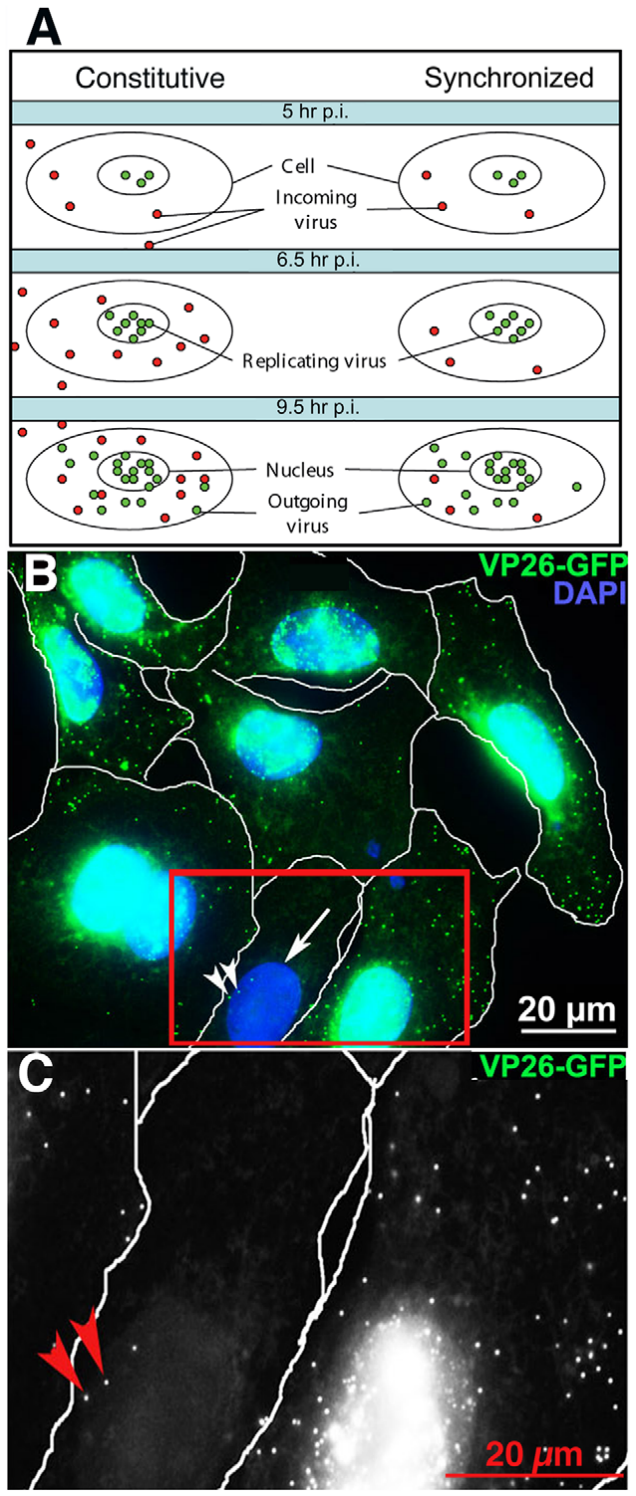
Synchronization of viral infection. (A) Diagram of the "in-coming/out-going" problem. Constitutive infections (left column), in which virus is added to the culture and allowed to remain throughout incubation, results in continuous entry of virus and mixing of in-coming and out-going viral particles in the cytoplasm. In synchronized infection (right column) viral exposure is limited by removal of the media, release of adherent virus from cell surface with acid-glycine and inactivation with human serum, resulting in few in-coming virus in the cytoplasm at later stages. Sub-confluent cultures are required to reduce viral transmission through cell-cell junctions. (B) An example of digital images used to quantify cellular locations of VP26-GFP particles. In this example, cells synchronously infected with VP26-GFP HSV1 (green) were fixed at 7 hr p.i., and counterstained with DAPI (blue). Images were collected by widefield fluorescence to detect VP26-GFP particles throughout the full thickness of the cells. Infected cell nuclei containing VP26-GFP appear turquoise at this exposure. Most of these infected cells display multiple viral particles in the cytoplasm. One cell (arrow) with no nuclear GFP fluorescence has three cytoplasmic GFP-particles (arrowheads) that therefore are in-coming virus. (C) Higher magnification of the boxed region in B in the GFP channel to show absence of diffuse nuclear fluorescence. Red arrows indicate two of the three GFP particles in this cell. See **Figure S1** for quantification.

We found that when confluent cell cultures are continuously exposed to virus, individual cells may acquire cytoplasmic viral particles from the media or through junctional contacts with other cells at any time during incubation. In such cultures individual viral particles in the cytoplasm of fixed cells cannot readily be identified as either incoming or outgoing. Since molecular motors mediating inward versus outward transport are different, the composition of incoming and outgoing viral particles must also differ. Without a reliable method to distinguish incoming from outgoing viral particles conclusions about the molecular composition of out-going particles is difficult. What is needed is a method to limit incoming particles. To do this we developed a natural yet rigorous infection protocol of sub-confluent cultures to limit in-coming virus within a short time period such that all infected cells were at a similar stage of infection. Since our goal was to image exiting viral particles, we did not aim for a uniform infection of all cells. We validated the protocol by counting VP26-GFP-labeled particles in the cytoplasm of infected cells at successive time points after various infection protocols (Figure 1 and Figure S1). Infected cells were identified by the presence of VP26-GFP in the nucleus, the source for out-going cytoplasmic viral particles. In productive infections, new capsids assemble first in the nucleus and then move outwards through the cytoplasm. Thus only cytoplasmic VP26-GFP particles in cells with nuclear VP26-GFP were considered potentially outgoing capsids. Cytoplasmic VP26-GFP particles in cells without nuclear GFP were considered incoming. The vast majority of cytoplasmic GFP-particles also stained for VP5 (92.8±2.9%, n = 2927), the major capsid protein, confirming that GFP-labeled particles primarily represent viral capsids (Figure S2).

While Campodelli-Fiume et al. reported that gD over-expression inhibits infection by measuring viral protein synthesis in cells expressing gD [33], both they and we observe viral particles in the cytoplasm of gD expressing cells [34]. Another group counted the number of viral particles stalled in the cytoplasm and correlated this with the genome/pfu ratios as an indirect measure of the number of defective particles in viral preparation that may enter a cell but not travel to the nucleus [31].

In a careful, quantitative study of various protocols previously used to block continuous viral entry, we found that none eliminated incoming viral particles in infected cell cytoplasm, including viral expression of gD or the presence of inhibitory human serum in the media. Since our focus was to witness HSV1-APP interactions in out-going cytoplasmic particles through immuno-staining and dynamic imaging, we concluded that reliance on gD expression or human serum to block re-infection was inadequate. We used a combination approach to limit infection to a narrow time frame (1 hr) as detailed in Materials and Methods. Quantitative analysis of cytoplasmic VP26-GFP-particles 4–5 hr after infection of sub-confluent cells with our synchronization protocol, a time point before emergence of newly synthesized capsids from the nucleus, revealed that in our synchronized infections only 3.3+/−2.3 VP26-GFP particles were present in the cytoplasm, indicating that on average 3 viral particles entered the cell or had been retained in the cytoplasm during 4–6 hr after the synchronized infection window (see Figure S1). This is equivalent to the best viral preparations previously reported [31]. We reasoned therefore that any particular cytoplasmic particle, even at later time points, in a cell with >30 particles in the cytoplasm has >90% chance of being out-going when cells are infected during a one-hour time window according to this synchronous infection protocol.

### HSV1 infection dramatically alters the distribution of cellular APP

**Figure 2:**
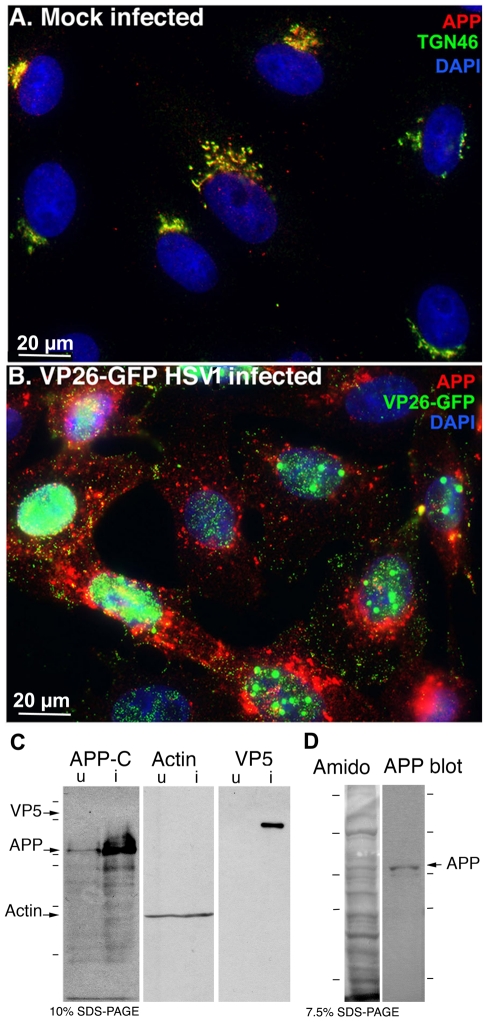
APP relocalizes in infected cells. (A) APP (red) co-localizes with TGN46 (green) in a compact tuft to one side of the nucleus (DAPI, blue) representing the trans-Golgi network in mock infected cells. (B) In cells infected with VP26-GFP HSV1 (green), APP (red) is distributed in particles throughout the cytoplasm at 7 hr p.i. Nuclei are stained with DAPI (blue). (C) Western blotting of uninfected (u) and infected (i) cells with anti-APP, anti-actin (loading control) and anti-VP5 (viral capsid) demonstrates significant increased amound of APP in infected cells. Actin bands remain similar, and VP5, as expected, is only detected in lanes loaded with infected cell lysate. Note no new APP bands are detected in the infected versus uninfected cell lysates. (D) Isolated virus separated on 7.5% SDS-PAGE and stained with amido black for protein and probed for APP by Western blotting with the same antibodies used for immuno-fluorescence. Note that only the 90–110 kD doublet representing APP is detected by anti-APP, with no additional viral protein bands detected. See also **Figure S2** for split channels and **Figure S3** for histone staining.

APP was visualized in mock-infected cells and in cells synchronously infected with VP26-GFP HSV1 by immuno-fluorescence (Figure 2). In uninfected cells, a tuft of APP staining to one side of the nucleus overlapped with TGN46, and thus likely represents the trans-Golgi network (Figure 2A) [35], [36]. In contrast, infected cells displayed APP staining throughout the cytoplasm by 7 hr p.i. (Figure 2B). Similar changes in staining patterns were seen in HSV1 infected human retinal epithelial cells (ARPE-19), Hela cells, and neuronal cell lines (PC12 and SHSY 5Y), although some cells retain the peri-nuclear position of the Golgi longer after infection than others. Redistribution of APP staining pattern was observed with three different anti-peptide C-APP^695^ antibodies from three different vendors (Sigma, against aa 676-695, Zymed against aa 673–695 and Chemicon against aa 686–695). The increased brightness of APP staining was confirmed by Western blot (Figure 2C) and is consistent with reports showing increased production of amyloid beta (A-beta), a product of APP, in HSV1 infected neurons [37], [38]. Since epithelial cells lack the enzymes to produce A-beta, excess APP expression that might be converted to A-beta in neurons would remain detectible as the full-length protein in epithelial cells. Notably, no additional bands are detected in Western blots of virally infected versus uninfected cells, demonstrating that the increase in APP antibody staining is not due to any anti-herpesvirus activity among the anti-APP antibodies. Finally we compared the ∼90–110 kD bands recognized by anti-APP with the protein banding pattern of isolated virus detected in a parallel lane of the same blot stained with amido black (Figure 2D). The bands migrating at ∼90–110 kD detected by anti-APP were similar to those in uninfected cells, and appear to correspond to the viral proteins, VP8–10, identified in metabolically labeled virions by Honess and Roizman [39].

HSV1 encodes an immunoglobulin Fc receptor, gE/gI, an envelope glycoprotein complex which binds the non-antigen-binding domain of immunoglobulin [40], [41], [42]. Like many viral proteins, gE may play more than one role– in addition to Fc binding, it appears to be required for anterograde transport of virus in neurons [43], [44], [45]. Although gE is classified as non-essential for growth in epithelial cells, deletions of gE do affect viral behavior in these cells: for example gE deletions result in smaller than normal plaques in epithelial monolayers [46], and display defects in transmission from infected epithelial cells to sensory neurons [44]. Since neurons and epithelial cells use similar machinery for transport, gE is also likely to be involved in intracellular transport in epithelial cells, although this process would not be essential to viral propagation in these cells, it may be responsible for smaller plaque size due to fewer virions reaching the cell surface for release.

To test whether the APP staining we observed was a result of specific antigen binding or an artifact of antibodies binding to the viral Fc receptor, gE/gI, we performed several control experiments: a) staining infected cells in parallel for APP and for a nuclear antigen, histone 3, using anti-histone rabbit antibodies of the same purity and at the same concentration as the anti-APP rabbit antibodies; b) staining cells infected with a virus deleted in gE; and, as shown later in this paper, c) knocking down APP with siRNA.

First, cells were stained in parallel for APP and for histones with rabbit primary antibodies and the same secondary antibody, all with similar purity and concentration, and imaged with the same camera settings. Viral particles were stained with anti-APP but not with anti-histone antibodies, which appropriately stained the nucleus predominantly (Figure S4).

**Figure 3:**
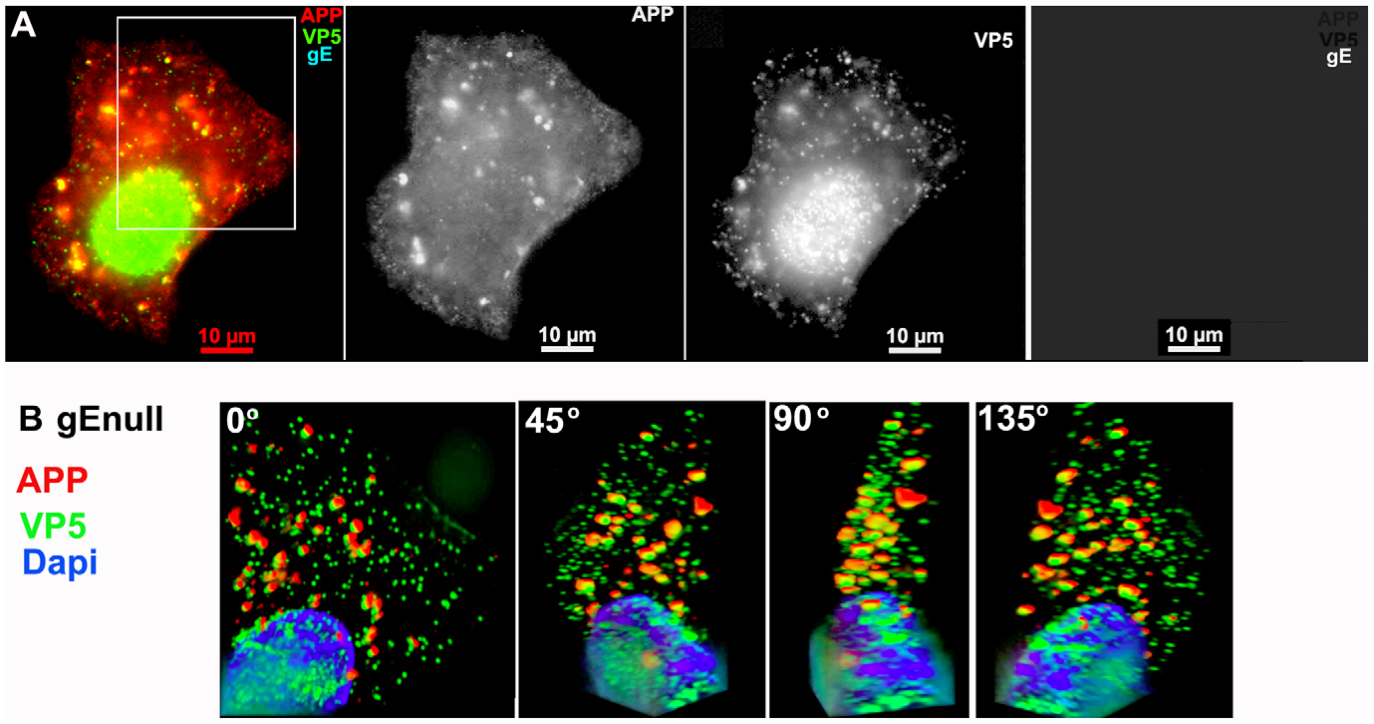
APP co-localizes with gEnull virus. A representative example of a cell infected with gE null virus stained for APP (red), gE (far red), and VP5 (green), the major capsid protein. (A) Low magnification wide-field image of the three channels, APP, VP5, and gE shown in color with merged channels (left), and then each individual channel, as labeled, of the same image. (B) Higher magnification of the boxed region shown in (A) and rendered in 3D. A Z-stack of 45 focal planes captured at 0.35 µm intervals was deconvolved using iterative processing and rendered in 3D. In four angles of rotation, co-localization of green VP5 capsids and red APP membranes throughout the cytoplasm of a cell infected with gEnull virus is shown. See **Movie S1** for a full rotation of the 3D stack shown in (B).

Next, we infected cells with HSV1 deleted in the gE gene, gEnull. Parallel cultures were infected with the parental strain, and the gE deletion virus with the gE gene re-inserted (gE rescue) [45]. The increased staining for APP and its redistribution after infection was still apparent in cells infected with gEnull virus. APP distribution in cells infected with either the parental strain or gE rescue infected cells was indistinguishable from that of cells infected with VP26-GFP virus (Figure 3A). In 3D images at higher magnifcation obtained by deconvolution of z-stacks, APP staining of gEnull viral particles was common among viral particles near the nucleus and less frequent in the peripheral cytoplasm (Figure 3B and Movie S1), an observation we will explore in more depth below.

### Cytoplasmic HSV1 particles co-localize preferentially with APP and with viral envelope glycoproteins and not with LAMP2, another cellular membrane glycoprotein, by immunofluorescence

**Figure 4:**
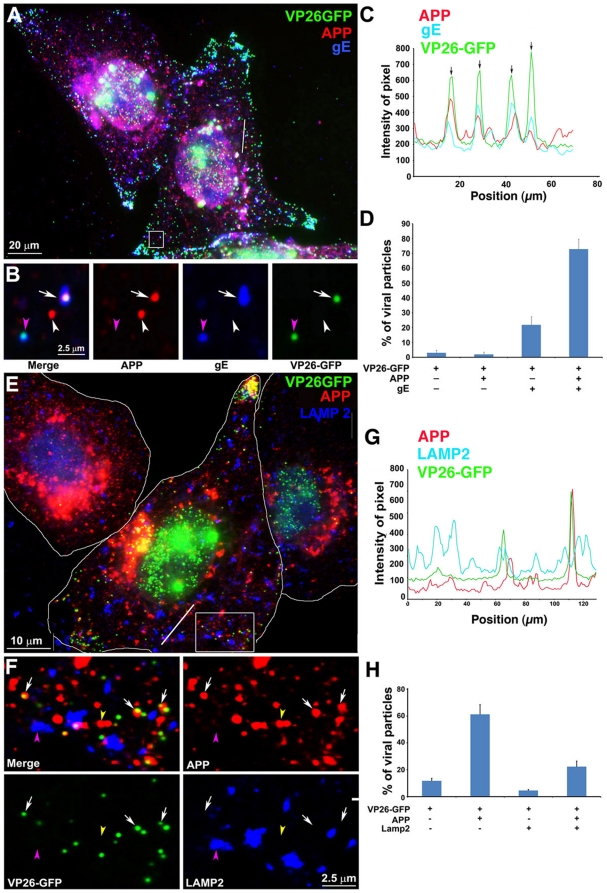
Out-going cytoplasmic VP26-GFP particles co-localize with APP and not with LAMP2. Cells were synchronously infected with VP26-GFP HSV1 (green) and fixed at 7 hr p.i.. (A–D) Examples of infected cells stained for gE (blue) and APP (red). In (B) a high magnification of boxed region in (A) is shown. A white arrow indicates one of the particles displaying all three labels (gE, APP and VP26). Particles with APP only (white arrowhead) or gE and VP26 (pink arrowhead) are indicated. (F–H) Examples of infected cells stained for LAMP2 (blue) and APP (red). In (F) a high magnification of the boxed regions in (E) is shown. White arrows indicate APP and gE, pink arrowheads indicate LAMP2, and yellow arrowheads indicate APP alone. (C and G) Intensity profiles along a line (white) drawn across the merged image in (A) and (E). Arrows indicate the superposition of peaks for each channel. (D and H) Histograms showing the percentage of VP26-GFP particles in each category. VP26-GFP alone (D: 3.0±1.7% and H: 11.8±3.9%) or with APP (D: 2.0±1.5% and H: 61.3±14.7%); VP26-GFP with gE (D: 21.8±5.5%) or with LAMP2 (H: 4.6±1.6%); and VP26-GFP with both APP and gE (D: 72.8±6.7%), or both APP and LAMP2 (H: 22.3±8.7%). Experiments were performed in triplicate, and 2085 particles in 11 cells were counted in D and 1592 particles in 9 cells in H. See **Figure S5** for triple label of gD instead of gE, with APP and VP26-GFP.

To characterize APP-capsid assemblies in more detail, we double-stained cells synchronously infected with VP26-GFP-HSV1 for APP and a viral envelope protein (either gE or gD) in cultures fixed at 7–9.5 hr p.i. (Figure 4 and Figure S5). The presence of a viral envelope protein together with the VP26-GFP-labeled capsid would support the notion that particles co-localizing with cellular APP were maturing viral particles undergoing secondary envelopment.

A high proportion of VP26-GFP particles throughout the cytplasm co-localized with both APP and gE: 72.8±6.7% (Figure 4A–D). VP26-GFP particles were rarely found without APP (3.0±1.7%). Thus co-localization with APP was statistically significant. Most importantly, one fifth of the VP26-GFP particles stained for gE but not for APP (21.8±5.5%), evidence that APP staining is not due to non-specific binding of the anti-APP antibody to gE via its Fc receptor function. Bleed-through between channels was undetectable (Figure 4B). Intensity profiles on a pixel-wise basis showed precise coincidence of intensity peaks for VP26-GFP with both APP and gE staining (Figure 4C). Viral gD displayed similar co-localization with GFP-particles and APP (Figure S5A–D). These results support other reports that viral capsids meet envelope in the cytoplasm during secondary envelopment when the envelope, with its glycoproteins, engulfs the particle [47], and extends these findings to identify a cellular membrane protein involved in this event.

At higher magnification with split channels VP26-GFP particles were uniform in size and shape, whereas particles stained for APP or either of the viral glycoproteins, gD or gE, varied in shape and size (Figure 4B). In some cases the APP stain surrounded the GFP-particle and in others the overlap with GFP-particle was partial, as if the capsid were attached to the surface of the vesicle or budding into it, likely reflecting the different capsid-membrane interactions seen by electron-microscopy [16], [19], [48].

Co-localization of viral particles with APP beyond the Golgi region was also biologically specific, since another organelle membrane protein, LAMP2, did not co-localize, except within the Golgi complex where membrane proteins are glycosylated (Figure 4E–H). LAMP2, a lysosome-associated membrane glycoprotein [49], co-localized with only a few GFP-particles in the cytoplasm beyond the peri-nuclear area (7.6±9.4%) (Figure 4E–H). GFP-particles stained for both APP and LAMP2 were only slightly more frequent (22.3±5.9%) (Figure 3H). This finding is similar to the sparse co-localization of HSV1 glycoproteins with LAMP1 previously reported [50], and demonstrates that co-localization of viral particles with APP is specific–i.e. antibodies to other cellular glycoproteins do not stain cytoplasmic viral particles.

### APP surrounds membranated viral particles by confocal and immuno-gold electron microscopy

**Figure 5:**
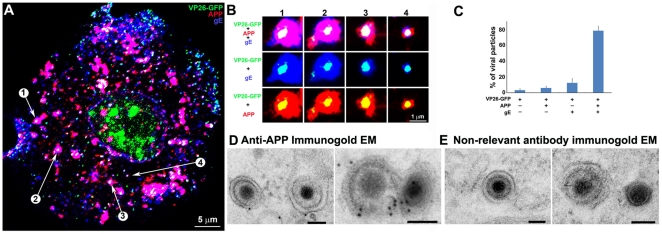
Confocal and immuno-gold electron microscopy demonstrate co-localization of both viral (gE) and cellular (APP) membrane proteins with VP26-GFP particles. (A) Example of a 0.8 µm optical section by confocal imaging of a cell infected with VP26-GFP HSV1 (green), fixed at 8.5 hr p.i., and stained for cellular APP (red) and viral glycoprotein, gE (blue). (B) Galleries of particles showing the co-localization of VP26-GFP with gE and APP. (C) Histogram showing the percentage of VP26-GFP particles in each category. VP26-GFP alone (3.2±2.0%), with APP (5.9±2.4%), with gE (12.4±5.6%) and with both APP and gE (78.4±5.7%). 569 particles in 5 cells were counted. (D) Thin section immunogold electron microscopy of HSV1 infected cells probed with anti-C-APP with protein-A linked 10 nm gold particles. Note single and multiple gold particles decorating membranes surrounding viral capsids in the cytoplasm. Bar = 100 nm. (E) Parallel sections from the same EM block treated with an irrelevant rabbit antibody of similar purity and dilution and probed with protein-A gold. Note the absence of gold labeling of viral particles. Also see **Figure S5** for co-localization of VP26-GFP particles with APP and viral protein gD, and **Figure S6** for additional immunogold electron micrographs.

First, to ensure that co-localization was not due to superimposition of separate particles from the full-thickness wide-field image, we captured images by confocal microscopy with 0.8 µm-thick optical sections (Figure 5). A gallery of images of individual particles shows GFP-particles singly or in clusters encircled by both viral envelope glycoproteins and APP (Figure 5B). Most VP26-GFP-particles are surrounded by membrane proteins as if inside a membrane compartment. Again, single-labeled VP26-GFP-labeled particles were rare (3.2±2.0%), while a high percentage of GFP-particles stained for both envelope glycoproteins and APP (78.4±5.7%) (Figure 5C). This confirms the co-localization seen in wide-field images, and provides additional detail of the APP-capsid interaction.

Co-localization of APP with viral particles was also detected at the ultrastructural level by immuno-gold thin section electron-microscopy (Figure 5D–E and Figure S6). Anti-APP antibodies were visualized by 10 nm protein A-gold particles in thin sections of infected cells where viral particles at various stages of maturation were clearly identifiable. Gold particles decorated membranated viral particles in the cytoplasm of infected cells, as well as membrane systems closely adjacent to viral particles, and both the clusters of viral particles inside larger membrane-bound organelles and the surrounding organelle membrane (Figure S6). Non-relevant polyclonal rabbit antibodies used in parallel on the same sections do not label intracellular HSV1 particles (Figure 5E), membranes in close proximity to viral particles, or clusters of viral particles within a larger membrane compartment (Figure S6). Immunogold labeling of uninfected cells was sparse, with only a few gold particles found within Golgi regions.

Thus, membranes containing cellular APP are physically associated with membranated cytoplasmic HSV1 at the ultrastructural level.

### siRNA knock-down of APP demonstrates specificity of APP-staining of peripheral viral particles

Anti-APP stained centrally located viral particles in both wildtype and gEnull HSV1-infected cells which demonstrated that this staining pattern was not a consequence of antibodies binding non-specifically to the viral Fc receptor, gE (Figures 2, 3, 4, 5). However, there was less APP staining of peripheral particles in gEnull-infected cells than in wildtype. This result could either be because there is some low level of antibody binding to the viral Fc receptor or because gE is required to retain APP-containing membranes during viral particle transit to the surface.

To distinguish between these possibilities we knocked down APP expression using siRNA. If viral particles expressing gE do not stain for anti-APP after APP knock-down, this would demonstrate that anti-APP label is not due to the viral Fc receptor, and suggest that gE may mediate retention of APP-containing membranes to emerging viral particles during their maturation and transit to the cell periphery.

**Figure 6:**
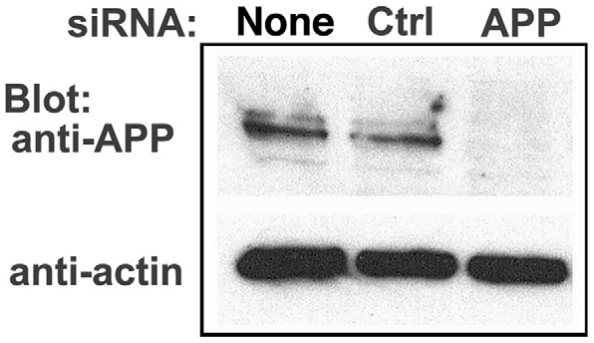
APP knock-down by siRNA decreases APP protein >90% by Western blotting. HSV1 infected cells were transfected in parallel with either vehicle alone (None), non-silencing RNA (Ctrl) or siRNA against APP (APP). After 48 hr cells were scraped into lysis buffer, and loaded in parallel on a 10% gel for electrophoresis followed by transfer to nitrocellulose. The blot was divided in two horizontally, the top half probed for APP and the lower half for actin, a loading control. Non-silencing siRNA has little effect, while siRNA for APP decreases APP band intensity almost entirely, with no significant effect on actin.

First, we confirmed knock-down by Western blotting in mock- and HSV1-infected cells, comparing no siRNA, non-silencing siRNA [51] or specific siRNA for human APP (Figure 6). In cells treated with the siRNA for APP there was a >95% decrease of the ∼90–110 kDa APP bands by Western blotting, while mock-infected and non-silencing siRNA retained similar band intensity for APP. Actin, a loading control, remains consistent across all 6 treatments.

**Figure 7:**
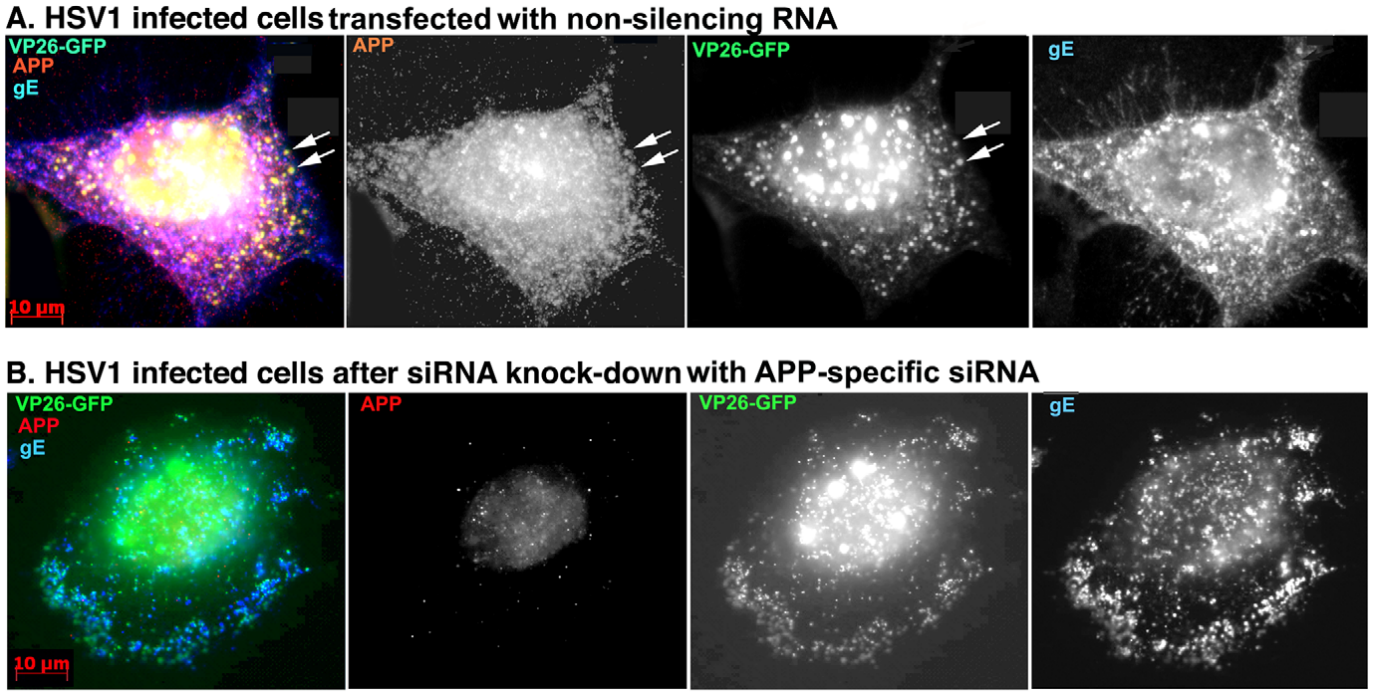
Knockdown of APP expression eliminates immuno-fluorescent staining of VP26-GFP particles by anti-APP antibodies. (A) Representative example of a cell treated with non-silencing control siRNA, infected with VP26-GFP HSV1 (green), fixed at 7 hr p.i. and stained for APP (red), gE (blue). APP staining is bright and diffuse, and co-localizes with VP26-GFP viral particles (arrows). (B) Representative example of a cell treated in parallel to the cell shown in (A) but with siRNA for APP. Note the absence of most APP staining, while gE staining of viral particles remains strong.

The immunofluorescence pattern of APP staining of VP26-GFP HSV1 infected cells after transfection with non-silencing siRNA was similar to untreated cells (Figure 7A). In contrast, cells transfected with APP-specific siRNA displayed no detectable staining of cytoplasmic viral particles despite an abundance of gE, which often co-localizedd with VP26-GFP particles (Figure 7B). Faint nuclear fluorescence of anti-APP staining in APP knocked-down cells most likely represents residual APP.

### Live imaging of capsid-APP dynamics

The results described above demonstrate a high frequency of interactions between intracellular APP and outgoing VP26-GFP-labeled viral particles. To discover whether interplay with APP makes a functional contribution to viral transport, we performed live confocal imaging of cells expressing mono-red-fluorescent-protein-labeled APP (APP-mRFP) infected with VP26-GFP-HSV1. Direct visualization of dynamic interactions does not require fixation and antibody staining, and also provides dynamic imaging of double-label viral particles. First, as a control, we confirmed that the mRFP signal represented APP by counter-staining fixed transfected/HSV1-infected cells with anti-APP antibodies. The majority (>95%) of mRFP particles also stained for APP by immunofluorescence (not shown), demonstrating that mRFP signal represents the APP-mRFP fusion protein.

**Figure 8:**
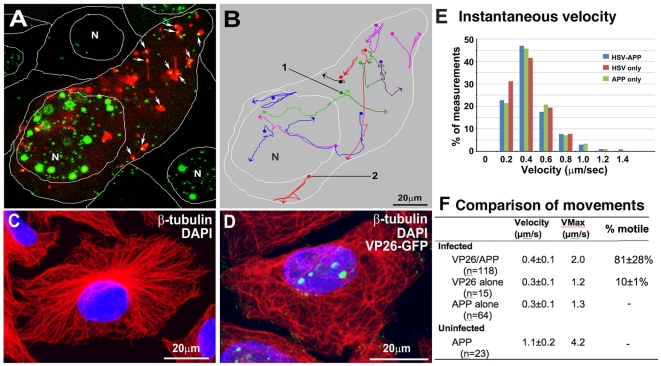
Co-transport of VP26-GFP particles and APP-mRFP in live cells. (A) The first frame of a video sequence captured at 7–9 hr p.i. captured at 3-sec intervals for 900 sec (15 min). Many (64%) VP26-GFP particles (green) co-localize with APP-mRFP compartments (red) in this frame appearing bright yellow (arrows). VP26-GFP particles travel with APP-mRFP vesicles, and sometimes join and separate from APP. White lines show the boundaries between cells and position of the nuclei (N). Also see **Movie S2**. (B) Tracings of some VP26-GFP particles moving with APP-mRFP. Traces are pseudocolored for ease of visualization. Dots and arrowheads indicate the beginning and end of movements, respectively. (C–D) Microtubules are in disarray in HSV1-infected cells. (C) Example of mock-infected and (D) VP26-GFP HSV1 infected cells stained for β-tubulin (red) and DAPI (blue) at 7–9 hr p.i. and imaged by laser scanning confocal. See also **Figure S7**. (E) Histogram of the instantaneous velocities in infected cells of dual VP26-GFP-APP, solo VP26-GFP and solo APP only particles. Instantaneous velocities of VP26-GFP-APP (n = 118), solo VP26-GFP (n = 15) and solo APP (n = 64) particles in 20 cells in 6 experiments were measured. (F) Table of movements. Quantitative comparison of average instantaneous velocity, maximum velocity and frequency of moves (% motile) for each type of particle from 3–5 different cells from 3 independent experiments, where n =  number particles measured. Note the decreased frequency of movements of the VP26-GFP particles without APP-mRFP, third column. See also **Movie S2**, **Figure S7 and S8,** and **Table S1**.

VP26-GFP particles were co-localized with APP-mRFP and the two labels moved together through the cytoplasm (Figure 8A–B and Movie S2). When VP26-GFP-labeled particles left the nucleus they were quickly met by APP-mRFP vesicles. In these flat cells (∼20 µm thick in our thin section electron-microscopy of scraped Vero cells), most particles moved in the x-y plane and could be followed until reaching the lateral borders [52]. On average, more than two-thirds of all VP26-GFP particles co-localized with mRFP during any 300-frame 10 min video sequence (70±21%).

The tracks followed by APP-VP26-GFP assemblies varied (Figure 8B). While some double-labeled particles moved smoothly in one direction, making steady progress towards the cell surface, others followed a zigzag pattern. This zigzagging contrasts with the linear movements of APP-YFP in neuronal processes [53]. Since active transport depends on microtubules, irregular tracks could be produced by viral-induced pathologic alterations in the underlying microtubule network [54]. Indeed, the microtubule network was altered after infection even at early time points (7 hr p.i.), as detected at low magnification (Figure 8C and D, and Figure S7). Mock-infected cells demonstrated the usual microtubule-organizing center (MTOC) located at one side of the nucleus with the typical spray of microtubules emanating from it towards the cortex (Figure 8C). In contrast, in HSV1-infected cells the MTOC was not identifiable, and the microtubule spray was disorganized with microtubules appearing curled, bundled, and lying both perpendicular and parallel to the cellular cortex (Figure 8D and Figure S7B and C). While the Lippe lab has reported that Golgi and microtubule stability in viral infection is variable [32], in these Vero cells this was not the case–all infected cells across the culture displayed microtubule disarray. By quantitative analysis of confocal imaging, many GFP-labeled particles (87% +/− 0.1%) were found adjacent to or touching microtubules (Figure S7C).

A functional link between APP-compartments and HSV1 became obvious when comparing the dynamics of VP26-GFP-particles with and without APP in cells expressing low levels of APP-mRFP. While velocities of VP26-GFP particles that moved were similar (Figure 8E), the propensity for a particle to move was much lower for VP26-GFP alone compared to VP26-GFP/APP-mRFP particles (Figure 8F). The majority of double-label particles (81±28%) traveled together through the cytoplasm for long distances (up to 60 µm, three times the diameter of the nucleus) (Figure 8F and Table S1). Some GFP-particles appeared to ride inside and some on the outside of motile APP-labeled membrane compartments. Some GFP particles joined, left, and re-joined these mRFP-labeled structures. In contrast to frequent movements of APP-VP26-particles, movements of VP26-GFP particles not associated with APP-mRFP were rare (10.1±1.1% motile, 88.9±1.1% immotile). Velocity of all particles that did move was similar, suggesting that similar transport machinery was in play.

While VP26-GFP particles traveled more frequently when associated with APP-mRFP, the opposite was the case for APP-mRFP particles, for which virus association decreased both velocity and propensity to move. Small APP-mRFP particles in uninfected cells moved at 1.1+/−0.2 µm/sec instantaneous velocity, while APP-mRFP particles in infected cells with or without viral particles moved more slowly (0.3+/−0.1 µm/sec) (see Figure 8E and F, Table S1). Hence infection with HSV1 and association of APP with viral particles affects the dynamic movements of emerging viral particles as well as of cellular APP-vesicles.

The velocity of APP/VP26 particles in the x-y plane suggests kinesin-driven transport, with the observed maximum velocity of 2.0 µm/s and average instantaneous velocity of 0.4±0.1 µm/s (n = 118 particles, from 20 cells in independent movies) (Figure 8E and Table S1).

**Figure 9:**
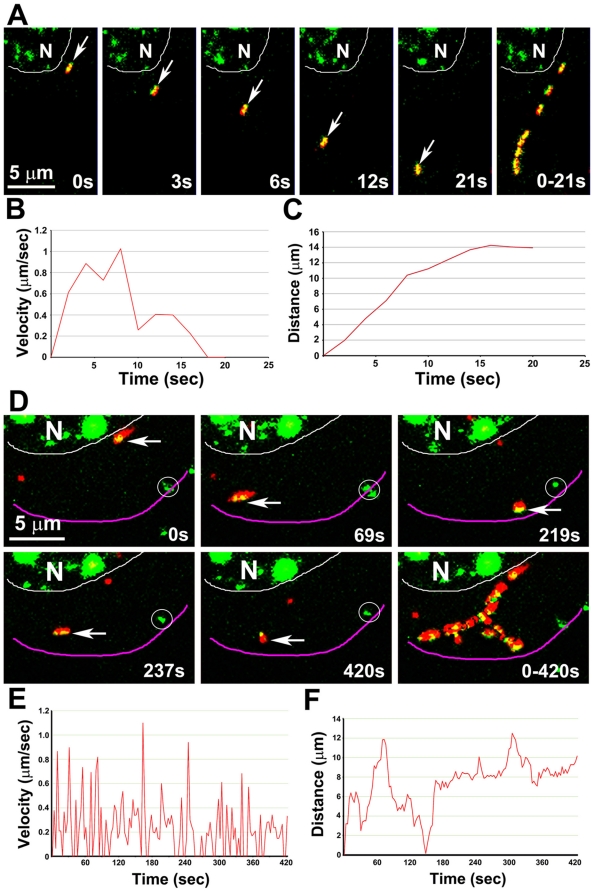
Representative movements of dual APP-mRFP and VP26-GFP particles. (A) A double labeled GFP-mRFP particle moves away from the nucleus (arrow, from **Movie S2** shown in Figure 5B, particle 1). The last panel shows 8 frames superimposed to demonstrate the pathway. See **Movie S3**. (B, C) Plots of velocity (B) and distance (C) versus time of particle 1 (in A). (D) A GFP-mRFP particle (arrow, from the video shown in Figure 5B, particle 2) moving away from the nucleus and ending at the periphery of the cell. Last panel shows 23 frames from a time-lapse sequence captured at 3-sec intervals (selected from a total of 141 frames of a 420 sec video) superimposed to demonstrate the pathway. The particle moves back and forth and along multiple tracks, and changes its shape during the movement. Circles indicate stationary GFP particles lacking APP-mRFP. See **Movie S4**. (E, F) Plots of velocity (E) and distance (F) versus time of particle 2 (in D), show the properties of the movements of each particle. Instantaneous velocity varies widely, with fast and slow velocities alternating.

When moving, instantaneous velocities of individual particles could be sustained (Figure 9A–B; See Movies S2 and S3) or fluctuate widely (Figure 9D–F; Movies S2 and S4). Motile double-label particles occasionally paused for long periods (24 sec) before resuming rapid movement (Table S1 and Figure S8A and Movie S4). Motile APP compartments sometimes changed shape during movement, adopting an elongated tubular shape while the VP26-GFP particle remained the same size, consistent with the interpretation that these GFP particles represent single capsids (Figure S8B; See Movie S5).

Live imaging of cells double-transfected with VP26-GFP and APP-mRFP and then infected with the gEnull virus demonstrated the same pattern seen by immuno-fluorescence (Movie S6). Cytoplasmic viral particles labeled with VP26-GFP did co-localize with APP in the nuclear area, while few viral particles were found in the peripheral cytoplasm. Little directed movement of single-labeled particles was seen, and rapid movements of double-labeled particles were also rare. Taken in the context of a requirement for gE in axonal transport of HSV1 and for accumulation of virus at the periphery of epithelial cells [20], [44], [45], [55], [56], [57], [58], and of our results in fixed epithelial cells infected with gEnull virus reported here, these live cell observations suggest a potential role for gE in viral transport within epithelial cells.

### Evidence for a specific interaction of HSV1 capsids with APP

We also tested the specificity of APP-capsid interactions by probing for another Golgi-enriched cellular protein, TGN46 [59]. If membranes containing APP randomly encountered capsids while passing through the trans-Golgi network, then we expected membranes with TGN46 would also meet capsids with similar frequency. Random co-localizations of APP, TGN46 and other secretory cellular proteins with nascent virus might occur in the trans-Golgi network where transport vesicles emerge for transit to the cell surface [15], [32], [60], [61], [62], [63], [64], since both cellular and viral glycoproteins pass through the Golgi apparatus for synthesis [65], [66]. Recent evidence that large particles such as chylomicrons also meet trans-Golgi membranes for transport implicates protein kinase D and a natural cellular secretory process in the packaging and transport of large viral capsids [67].

**Figure 10:**
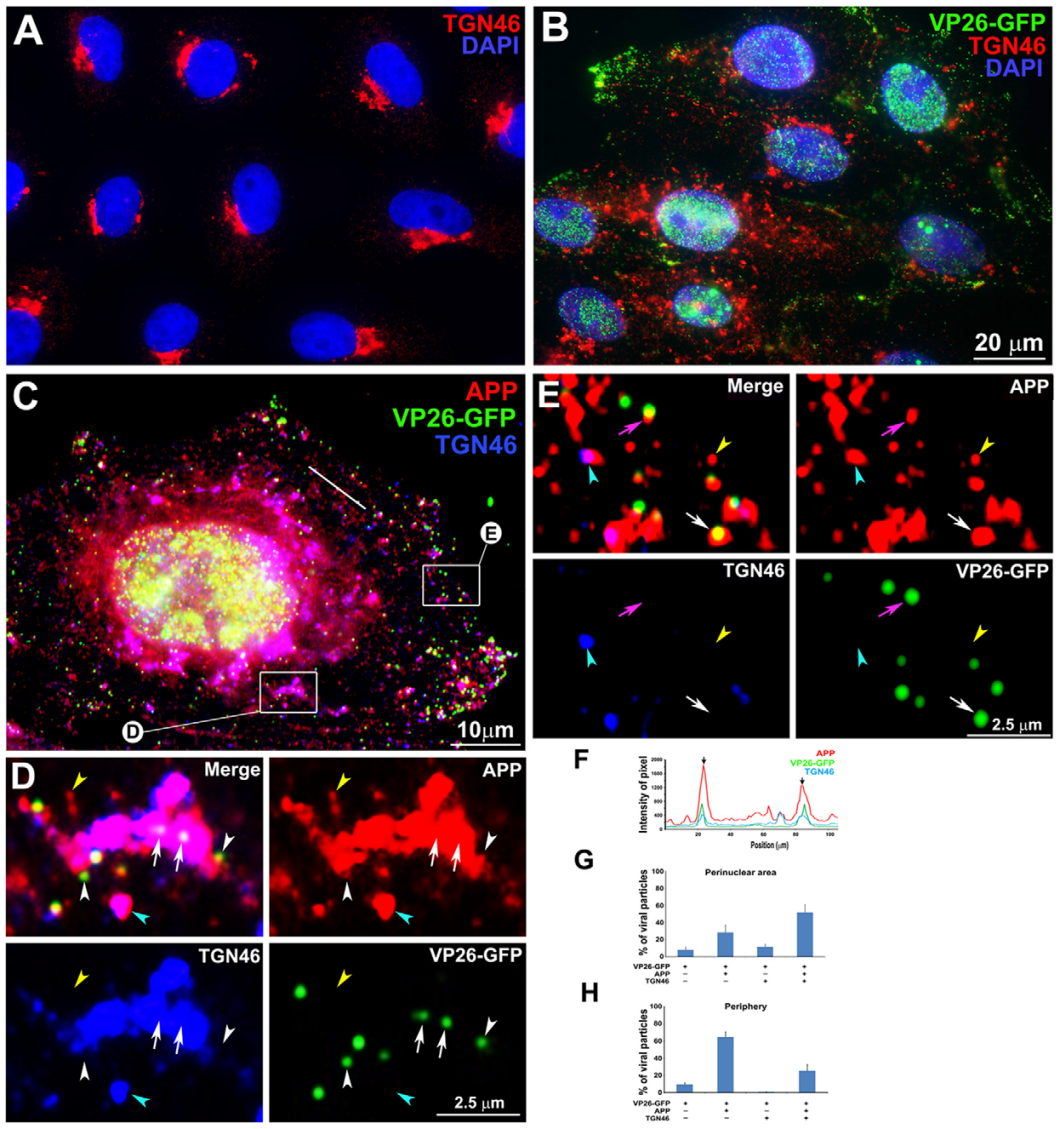
APP and TGN46 do not co-localize with VP26-GFP labeled viral particles in the periphery of HSV1 infected cells: Evidence for specific APP-HSV1 interactions. (A) Uninfected cells stained for TGN46 (red) show compact tufts on one side of the DAPI-stained nuclei (blue). (B) Cells synchronously infected with VP26-GFP HSV1 (green), fixed and stained for TGN46 (red) and DAPI for nuclei (blue). (C) Higher magnification of an example of cell infected with VP26-GFP-HSV1 (green) fixed at 7 h p.i. and stained for TGN46 (blue) and APP (red). (D) Co-localization in the peri-nuclear region shown at high magnification of boxed region "D" in cell shown in (C). Arrows indicate co-localization of VP26-GFP particles with APP in a TGN46-stained compartment. White arrowhead indicates a VP26-GFP particle apparently on the surface of a TGN46-stained vesicle. Yellow and cyan arrowheads indicate examples of single anti-APP-stained and both APP and TGN46 stained particles, respectively. (E) Loss of co-localization at the periphery is shown at high magnification of boxed region E at the periphery of the cell shown in (C). Pink and white arrows indicate co-localization of VP26-GFP HSV1 with APP alone at the periphery and in the cytoplasm close to the periphery, respectively. Yellow and cyan arrowheads indicate examples of single APP and both APP and TGN46 labels, respectively. (F) Linescan intensity profile of a region in the intermediate cytoplasm as seen in (C) shows both coincident (arrows) and non-coincident (arrowheads) peaks of TGN46 staining (blue line) with APP (red) and VP26-GFP particles (green). (G) Histogram of particles in the peri-nuclear region showing the percentage of VP26-GFP particles that co-localized with APP and TGN46. The majority of VP26-GFP particles co-localized with APP and TGN46 (51.8±9.5%) and fewer co-localized only with TGN46 without APP (11.6±3.1%). (H) Histogram of particles in the periphery showing the percentage of VP26-GFP particles that co-localized with APP and TGN46. None co-localized with TGN46 alone, although 25.5±14.2% were co-localized with both APP and TGN46. Note that many fewer particles co-localized with TGN46 in the periphery than in the peri-nuclear region, suggesting that membrane compartments co-localized with viral products retain the ability to sort their components. N = 10 cells, 3,782 particles from three experiments.

As for APP, TGN46 also moved from its normal peri-nuclear location to become distributed throughout the cytoplasm by 7 hr p.i. (Figure 10A and B). In regions close to the nucleus, most VP26-GFP particles appeared inside larger TGN46-staining structures that also stained for APP (Figure 10C and D). Quantitative analysis revealed that the majority of peri-nuclear VP26-GFP particles co-localized with both TGN46 and APP (53.2±9.8%), and fewer with APP alone (27.4±8.2%) (Figure 10D and G). Intensity profiling showed co-alignment on a pixel-wise basis of all three fluorescent particles in the peri-nuclear region (Fig. 10F).

In sharp contrast, in the peripheral cytoplasm many VP26-GFP particles co-localized with APP but very few also had TGN46 staining (Figure 10C and E). At these more peripheral locations, only 25.5±7.5% of VP26-GFP particles co-localized with both TGN46 and APP, while 64.7±8.8% co-localized with APP alone (Figure 10E and H). Notably no VP26-GFP particles in either location were found to co-localize with TGN46 alone. These data are explained if interplay between capsid and APP is not random, is more robust than with TGN46, and if Golgi elements were able to maintain some level of sorting of transport vesicle proteins despite viral occupants.

At slightly later times (9 hr p.i.) the large triple-labeled clusters in the Golgi region seen at 7 hr p.i. were less pronounced, and some appeared to have drifted outwards to the intermediate cytoplasm. This change may reflect the fragmentation of the Golgi that occurs in HSV1-infected Vero cells [48], [68]; the re-organization of microtubules shown here and reported elsewhere [54], [69]; and the redistribution of the trans-Golgi network marker, TGN46, reported to occur in confluent epithelial cell cultures after 12 hr of continuous exposure to herpes virus [50], [70]. This fragmentation of the Golgi and reorganization of the microtubules induced by HSV1 infection affects the critical location of APP. Interactions of viral particles with APP-containing membrane systems may contribute to Golgi fragmentation and microtubule instability, possibly by altering the distribution and/or function of the cellular transport machinery and its various cargos.

## Discussion

By synchronizing infection and performing live imaging of VP26-GFP-labeled capsids in cells expressing fluorescently labeled APP, we report results that provide new insights into the interaction between viral and host cell components during transit of virus to the surface. Functional relevance to the virus of interplay with APP, an identified motor receptor, is clear: association of viral capsids with membrane systems containing APP confers a propensity to move through the cytoplasm at fast-anterograde transport rates. Riding on or entering cellular membrane systems that normally traffic to the surface would thus promote efficient viral egress. By coordinating secondary envelopment with acquisition of the cellular vesicular transport machinery, the virus would be assured of reaching the cell surface in an infectious form.

In summary, the evidence includes: (1) VP26-GFP-labeled particles in the cytoplasm are frequently found together with both viral glycoproteins and cellular APP; (2) VP26-GFP-labeled particles travel together with APP for long, rapid trajectories, and GFP particles lacking APP move less often; (3) Co-localization with APP is specific, since GFP-labeled capsids and viral glycoproteins were less frequently found with other cellular organelle membrane proteins, LAMP2 and TGN46; (4) gEnull particles stain for APP, and siRNA knock-down of APP abolishes staining; and (5) VP26-GFP particles sustain co-localization with APP-mRFP throughout transport, while by immuno-fluorescence co-localization of VP26-GFP with TGN46 is lost. Thus sorting of cargo is preserved in HSV1 infected cells at least at these time points after infection, and VP26-GFP-labeled particles appear to interact with a select APP-containing Golgi-derived membrane compartment.

Interaction of viral particles with APP-containing membranes is not without functional consequence: APP-mRFP particles travel more slowly in infected than in un-infected cells, even without detectible viral cargo, and APP is mis-localized in HSV1-infected cells. Such mis-localization could contribute to increased APP proteolysis with HSV1 infection [37] and lead to additional, as yet unrecognized, HSV1-induced cellular injury.

Collectively, our results provide new information about dynamic interactions between nascent viral particles and cellular membranes, the molecular composition of virus during outbound transport, and suggest how secondary envelopment and transport to the surface may be coordinated. Such dynamic interactions between APP and HSV1 suggest a mechanistic basis for the observed clinical relationship between HSV1 seropositivity and risk of Alzheimer's disease [71].

### Synchronization of infection improves identification of out-going viral particles

By synchronizing infection we minimized viral re-entry during replication, ensuring that at later time points during productive infections, >90% of cytoplasmic viral particles are progeny undergoing outbound transport. Other methods to synchronize infections include the use of mutant viruses, temperature shifts, and pharmacologic interference with synthetic pathways [67]. We chose here to apply a natural, non-mutation or drug-based method to synchronize infection so as to interfere as little as possible with the basic, as yet unknown, mechanisms for packaging and transport. Thus hopefully these methods will also enable future discovery of molecules that differ between in-coming and out-going virus under natural conditions.

### Outgoing capsids travel with APP

**Figure 11:**
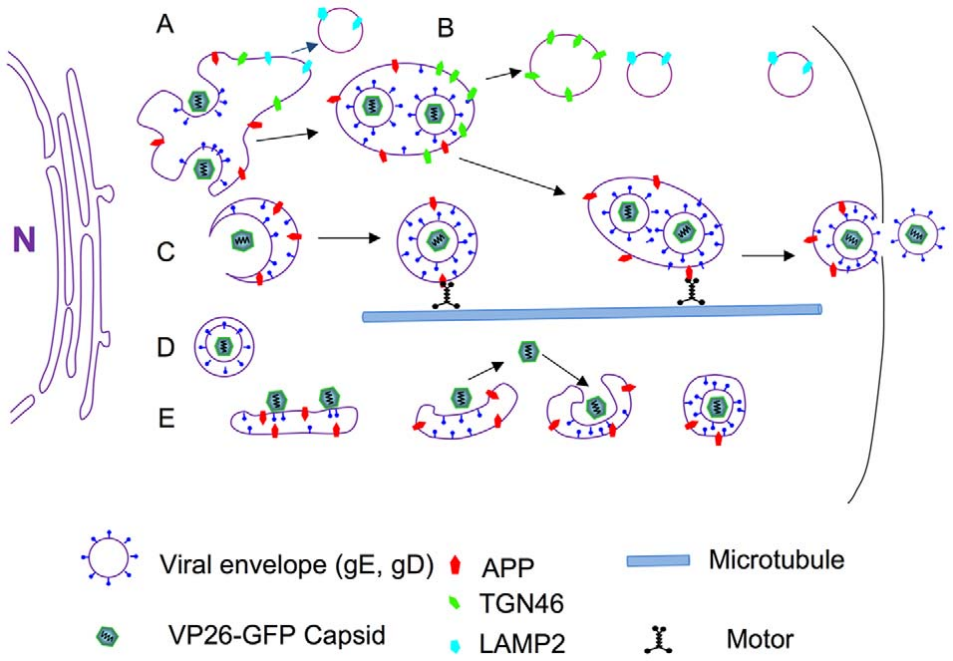
Diagram. A cartoon showing various types of interactions between cellular APP and VP26-GFP labeled viral particles documented here. (A) In the peri-nuclear region, VP26-GFP particles dance around and within large peri-nuclear compartments co-localizedd with viral envelope proteins, gE and gD, and cellular membrane proteins, LAMP2, TGN46 and APP. LAMP2 compartments separate from this apparent Golgi network early and rarely co-localize with viral components at the periphery. Some membrane systems with VP26-GFP also label for both APP and TGN46, primarily near the nucleus at the time points studied here. (B) TGN46 particles separate from VP26-GFP labeled viral components farther towards the periphery, while the APP particles remain with VP26-GFP particles and with viral envelope glycoproteins, gE and gD, en route towards the cell surface. (C) VP26-GFP particles may enter smaller post-Golgi APP-staining particles that undergo directed transport. (D) Some VP26-GFP particles remain separate from APP after leaving the nucleus. These may be inside unlabelled membrane systems or be free in the cytoplasm, some have the capacity to transport without APP. (E) VP26-GFP particles may ride on the cytoplasmic surface of APP-labeled membrane systems, come on or off these membranes, or bud into them. Any particular viral particle may employ all of these mechanisms during transit in the cytoplasm. In each case, we hypothesize that microtubule motors, such as kinesin, are recruited, possibly via APP or another cellular motor receptor.

Models for the location of envelopment and the molecular composition of viral particles transporting to the cell surface are quite varied and a topic of considerable debate. Two possibilities for capsid transport are proposed: independent or membrane-associated (see diagram, Figure 11). In our model, as demonstrated here, we observe the following: At early time points during viral production, VP26-GFP labeled capsids recruit APP during secondary envelopment from cellular membrane systems, principally the trans Golgi network, and possibly also recycling endosomes, which likely assume a more prominent role at later time points after infection. Others have reported that entry of capsids into the apical side of the TGN may follow the alternate pathway for trafficking of large particles such as procollagen and chylomicrons [67]. The budding of alpha herpesvirus into cellular membrane systems may be similar to mechanisms by which cellular vesicles enter into multivesicular bodies via ESCRT proteins [72]. Indeed, isolated enveloped virus contained within a second cellular-derived membrane transports on microtubules *in vitro* [73].

In our videos it sometimes also appears that the VP26-GFP particle is riding on the cytoplasmic surface of cellular membranes that are undergoing transport. These ideas as well as other possible transport configurations are diagrammed in Figure 11.

Budding into dynamic vesicles may be critical for nascent viral particles to acquire anterograde motors, such as the kinesins, for transport to the surface. When co-localized with an APP membrane, VP26-GFP particles moved significantly more frequently and at higher velocities. Thus our live imaging of cellular APP and viral capsid reveals a functional link between them: APP-containing membrane alliance confers efficient motility to the virus.

### Evidence for non-enveloped transport

Evidence that the other model of non-membrane-associated transport occurs is also presented here (see Figure 11) [4], [6], [10], [18], [20], [43], [74], [75], [76]. VP26-GFP particles traveled both with and without APP, although those without detectible APP were in the minority at the early time points after infection reported here. Thus capsid/tegument complexes may interact directly with cellular motors during egress as they do upon entering the cell [77], [78]. Our previous work showed that capsid-tegument complexes isolated by detergent treatment of infectious particles transport uniquely retrograde when injected into the squid giant axon [79]. Since then the tegument components involved were elegantly identified in *in vitro* motility assays [80] and later found to bind both plus- and minus-end directed motors in reconstitution assays [78].

Reorganization of microtubules in HSV1-infected cells must play a large role in these two mechanisms of viral transport. We show here that the microtubule organizing center (MTOC) is lost in infected Vero cells, and microtubules project from the full circumference of the nucleus as has been previous described [54], [68]. This reorganization may in part be due to ICP0, a viral protein that dismantles the microtubule network independent of infection, inducing coarse nets of aggregated tubules [81].

At later stages, the large triple-labeled clusters of capsid-envelope-APP, initially found in the peri-nuclear region, became less pronounced and some clusters appear to move outwards to the intermediate cytoplasm, probably a consequence of microtubule reorganization. Thus as productive infection proceeds, capsids must travel farther after exiting the nucleus to reach membrane compartments for envelopment. This observation indirectly supports a necessary role for membrane-free capsid transport from the nucleus to cortical membrane compartments for envelopment. Reorganization of the microtubules may allow retrograde motors to carry nascent viral capsid-tegument assemblies from the perinucleay area to the cortical Golgi at late time points of productive infections in epithelial cells.

### Intracellular versus extracellular virus

Our data show APP-containing membranes travel together with intracellular HSV1 particles, and contrast with a failure of mass spectroscopy to detect APP in preparations of extracellular HSV1 virions [23]. What might be the basis for this discrepancy? One obvious difference is the type of particle studied: intracellular viral particles versus extracellular virus. Thus HSV1 may lose APP membranes upon release from the cell, as would happen if APP were in a second cellular membrane encircling intracellular viral particles and not in the viral envelope. Indeed, such a second membrane was found in our study here, and has been elegantly shown by electron-microscopy of both intracellular HSV1 and PRV, a related alpha herpesvirus [9], [15].

### How HSV1 travels outwards in epithelial cells

Synthesizing our results with the work of others, we propose various models for coordination of capsid envelopment and transport to the cell surface after emergence from the nucleus (Figure 11). In one scenario, capsids exiting the nucleus at early time points after viral replication meet Golgi elements in the peri-nuclear region, where both viral and cellular membrane proteins are glycosylated. Capsids would thus acquire envelope by budding into Golgi compartments and entering cellular compartments whose membranes contain APP, a vesicular motor receptor that binds kinesin-1 [82] and drives anterograde transport [27]. Alternatively, nascent capsids may enter cellular transport vesicles in the trans-Golgi network or even during their transit through intermediate cytoplasm. Capsids lacking APP may be either naked in the cytoplasm, complexed with tegument, or associated with an unlabeled membrane compartment. Since some of these move, they must have alternative mechanisms to attract motors, such as tegument recruitment of kinesin and dynein [78]. Finally, nascent viral particles may ride on transport vesicles, possibly also coming in and out of the vesicle en route to the synapse or cell surface.

### A role for HSV1 in APP dynamics

Emerging **e**pidemiological evidence links cognitive impairment with peaks in anti-HSV1 antibody titers in human serum [71]. More recently, chronic active HSV1 infection in the central nervous system has been reported [83], lending additional credence to the idea that chronic active CNS infection may last late in life undetected, causing progressive damage. Correlations at the molecular level between genes associated with AD and cellular proteins involved in HSV1 infection are described, although direct experimental evidence is still lacking [84]. Here we show that HSV1-infected cells display abnormal APP distribution, and APP and HSV1 capsids co-localize and travel together within cells, apparently by fast anterograde transport mechanisms. Thus cellular APP is affected by HSV1 infection–its distribution, its dynamic movements, and possibly its post-translational modifications, including phosphorylations and proteolysis [37]. HSV1 DNA is found in senile plaques [85]. While a role for HSV1 in AD remains controversial, the notion is especially intriguing since HSV1 infection offers a potentially treatable target.

Once inside the neuron, HSV1 enters a latent stage that persists for the lifetime of the host. Decline in immunity associated with aging {Arlt, 2004 #292;Kurz, 2004 #291} increases chronic reactivation of herpesvirus [86]. Once A-beta has accumulated beyond a particular point, removal becomes difficult and repeated deposition results in more, larger plaques. Circulatory changes with decreasing perfusion, also correlated with aging, would compound this problem, since A-beta is digested and removed by serum enzymes. Reactivation of virus results in the recurrent "cold" sore on the lip. Such long-term chronic infection may not be benign. Since the concept of slow viral disease first proposed by Gajdusek [87], many examples have been found. Famous among these are HPV [88], HIV [89], as well as another member of the herpesvirus family, Epstein-Barr virus [90]. Hence the question is not *whether* HSV1 causes neurological damage, but rather *how significant* that damage is over a lifetime. Nascent virus induces a profound alteration in cellular membrane organization and anterograde transport, subcellular systems known to be required for neuronal function and survival. The interactions of HSV1 with APP shown here suggest a molecular mechanism for the observed epidemiological correlation: long-term chronic active HSV1 infection would affect APP distribution, expression and processing, and thereby have significantly more impact than previously considered.

## Materials and Methods

### Virus and cell culture

VP26-GFP HSV1 was a gift from Dr. Prashant Desai (Johns Hopkins University) [28]. VP26-GFP HSV1 expresses green fluorescent protein (GFP)-fused inside the first 4 amino acids at the N-terminus of UL35 (VP26), a 12 kDa minor capsid protein [29], [30]. Viral strains with the gE gene knocked out, the parent strain (NS) and a strain created from the gE null with the viral gene replaced, were obtained from Harvey Friedman [45], [91] and the knock-out gene replacement confirmed by sequencing after propagation in the Bearer lab. Vero, HELA, and ARPE-19 cells (ATCC) were cultured on glass coverslips or chambered glass coverslips (Lab-TeK) in Dulbecco's Modified Eagle's Medium (DMEM) supplemented with 10% fetal bovine serum and used for 25 passages.

Virus was isolated as described [79]. Basically, confluent Vero cells are infected with a viral stock at ∼1–10 MOI and incubated at 37°C. Cells are harvested when they began to round (16–17 hr p.i.) by scraping together into the media, transferred to a 50 ml sterile tube and freeze-thawed 3 times by passing the tube from dry ice-ethanol (−78°C) to 37°C water bath. Viral particles in the cytoplasm are released from cells by low-energy sonication (Fisher Scientific 550 Sonic Dismembrator) set at 3.5 for 10–15 sec. The viral solution is then centrifuged for 10 min at 2000 rpm (1000×g) in an IEC serofuge at 4°C to remove nuclei and cellular debris. After filtration through a 0.45 µm Millipore filter, the supernatant is layered over a discontinuous sucrose gradient (3 ml of 10%, 8 ml 30%, 8 ml 60% in PBS) in 38.5 ml disposable centrifuge tubes for the SW28 rotor (Beckman) and centrifuged for 2 hr at 25,000 rpm (80,000×g). The lower fuzzy white band, just above the 60–30% sucrose interface, is collected (2–3 ml), transferred to a fresh centrifuge tube and diluted to 30 ml with PBS. Diluted virus is centrifuged again at 25,000 rpm in the SW28 rotor for 1.5 hr at 4°C, the resultant pellet layered with 150 µl of PBS and left overnight to resuspend without mechanical mixing. The 150 µl viral suspension is then aliquoted, drop-frozen in liquid nitrogen and stored at −80°C. This yields viral preparations with 2–30×10^9^ pfu/ml and genome/pfu ratios of 23–98 without and 2.5–46 with DNAse treatment using the protocol described in [31], and also see below. Negative-stain electron-microscopy demonstrated that 49–50% of viral particles retain viral envelopes as we previously described [17], [79].

### Measurements of genome to plaque-forming unit ratios

The number of viral genomes was determined in parallel by quantitative PCR both with and without DNAse treatment, using the gB primers as described [31]. Briefly 2 ul of gradient-purified virus is mixed with 400 µl of 1× reaction buffer (10 x DNase 1 reaction buffer: 100 mM Tris-HCl, pH 7.5, 10 mM MgCl_2_ 5 mM CaCl_2_ (Ambion)) and then split into two 200 µl aliquots. One aliquot is treated with 2.5 µl of DNase1 (5 units: Abgene recombinant DNase 1, Thermofisher AB) and both tubes incubated at 37°C for 30 min and then at 75°C for 10 min. After incubation, 2.5 µl (25 mg total) salmon sperm DNA (Invitrogen) is added to each tube. DNA is then extracted with Qiagen mini kit for a final volume of 200 µl. Samples are then diluted 1∶10^−3^ and 1∶10^−4^ in reaction buffer producing a DNA preparation ready for PCR quantification.

Quantification was determined by comparison of the DNA from our viral prep to known concentrations of genomes obtained commercially for clinical studies, HSV1 Type 1 DNA Macintyre strain (Advanced Biotechnologies Inc.) run in parallel. Viral preps and these standards with and without DNAse treatment are mixed with 12.5 µl SYBR Green Master Mix (Applied Biosystems Inc), 25 mM of each forward and reverse sense primer and 5 µl of diH_2_O. Primers were selected from the HSV1 glycoprotein B, an essential gene. The forward sense primer is 5′-CCA CGA GAC CGA CAT GGA GC-3′ and reverse primer 5′-GTG CTY GGT GTG CGA CCC CTC-3′ [25] resulting in a predicted 246 base-pair product from HSV1 viral genomes according to the database of HSV1 F-strain, NCBI Ass. Number GU734771.1. After one cycle of denaturation (95°C for 10 min), 40 cycles of amplification are performed (95°C 10 s and 60°C for 1 min). During each 60°C phase fluorescence is measured and from this data the DNA concentrations are calculated against a standard curve generated from the series of dilutions of known viral copy numbers run in parallel.

To determine plaque-forming units, viral preparations were titered in triplicate by infecting confluent Vero cells under synchronizing conditions. Vero cells were plated at 0.5×10^6^ cells per 6-well plate and incubated overnight at 1.5×10^6^ confluent cells per well after division. Gradient-purified virus was serially diluted in growth media (1/100 to 1/10^−8^ viral preparation/media). Each viral dilution (0.5 ml) was inoculated into three wells of confluent cells on ice. After an hour cultures were warmed to 37°C and incubated another hour and then rinsed in acid-glycine according to the synchronization protocol (below). To limit subsequent infection through the media, cultures were overlaid with 0.5% agarose in growth media plus 1% human serum (Gibco/invitrogen) after the acid-glycine rinse. Thus virus has 1 hr to enter cells. Cultures were incubated at 37°C for 2–3 days when plaques become distinctly visible.

### Plasmids

A pMonoRed-APP695 plasmid encoding MonoRed (mRFP)-tagged 695-aa human APP (APP695) was generated from a backbone plasmid, pEGFP-N3/VP16 [79], by replacing GFP with mRFP amplified from mRFP1-pRSET-b plasmid (from Dr. Roger Tsien) [92] and replacing VP16 with APP695 from the APP-YFP-coding domain in pShuttleCMV plasmid (from Drs. E.-M. Mandelkow and Jacek Biernat, Max Planck Institute, Hamburg, Germany) [53]. MonoRed (mRFP1) sequence was amplified by PCR using BamHI forward, 5′-CGGGATCCATGGCCTCCTCCGAGGAC-3′; NotI Reverse, 5′-TTGCGGCCGCTTAGGCGCCGGTGGAGTG-3′ primers and ligated between the BamH1/Not1 site in pEGFP-N3/VP16. This generated a new plasmid, pVP16-MonoRed, with mRFP at the carboxyl terminus of VP16. APP695 was amplified from human APP-YFP in pShuttleCMV with the primers: HindIII Forward, 5′-GCAAGCTTATGCTGCCCGGTTTG-3′; SacII Reverse, 5′-CGCCGCGGGGTTCTGCATCTGCTCAAAGA-3′ and ligated between the HindIII/SacII sites in pVP16-MonoRed. Thus, mRFP tag is located 18 nucleotides (CCC GCG GGC CCG GGA TCC) from the last nucleotide coding the COOH terminus of APP. Expression from this plasmid is driven by the CMV promoter. The sequence of APP695 in pMonoRed-APP695 plasmid was verified. The VP26-GFP plasmid, pK26GFP, was obtained from Desai [28]. Expression from this plasmid is driven by UL35 promoter.

### Natural Synchronization of Viral infection

To synchronize viral infection, subconfluent cultures were first chilled on ice for 30 min-1 hr and then inoculated with VP26-GFP HSV1 virus, typically diluted to a viral concentration of 10 plaque-forming units per cell, i.e.10 MOI. We used subconfluent monolayers because our live video experiments had revealed viral particles passing through junctions between cells in confluent cultures (Bearer and Ferland, MS in preparation). Because our goal was to create culture conditions that limited in-coming virus without resorting to genetic manipulation of the virus or pharmacologic intervention, we used sub-confluent monolayers to reduce such junctional transfer of virus between cells, and short (1 hr) infection times. Even at 10 MOI with a 2.5 genome/PFU ratio, this did not result in infection of all cells. We accepted this lower infection efficiency in favor of better assurance that the possibility of in-coming virus at later time points was eliminated to the extent possible. Even though the virus was titered under synchronizing conditions, that titering requires confluent monolayers, where each virion has a better chance of encountering a cell. In subconfluent cultures, with cells not touching each other, virions also fall between cells necessitating higher viral MOIs for efficient infection rates.

After inoculation, cells were incubated on ice for another 30 min-1 hr to allow virus to adhere, and then warmed to 37°C to allow viral entry. After 1 hr infected cells were washed with acid glycine for 3 min at RT (0.14 M NaCl, 5 mM KCl, 1 mM MgCl_2_-6H_2_O, 0.7 mM CaCl-2 H_2_O, 0.1 M glycine, [pH 3]) [93] to remove adherent virus and any viral particles in the media. Low pH is reported to bypass the gD-inhibition and drive virus into cells in a gD independent manner [94], [95], [96]. To prevent re-entry of emerging virus later in infection and block any residual virus remaining in the culture from initial inoculations, 1% human serum, containing anti-HSV1 antibodies that neutralize extracellular virus, was added [97]. To our knowledge, we are the first to use this combination of low temperature inoculation to maximize virus-cell adhesion without internalization, low pH wash to induce simultaneous entry or release of adherent virus, and neutralizing antibodies to control the timing of infection. The infected cells were then fixed at 6, 7 and 9.5 hr p.i. and stained with DAPI. For quantification, images of fields of infected and mock-infected cells were randomly selected. We used widefield for a comprehensive picture of the full-thickness of the cell. The flatness of Vero cells allowed us to focus on capsids throughout the cytoplasm with wide-field illumination. From the collected images, 16,300 capsids from 165 infected cells were counted and categorized according to number and location, cytoplasmic or nuclear. We did not attempt to quantify nuclei with more than 10 capsids as the GFP fluorescence demonstrated that these were clearly highly productive of viral particles. GFP particles in the cytoplasm were clearly distinguishable from aggregates and rare GFP debris as capsids have a uniform size (See Figure S2).

### Immunofluorescence

Cells on coverslips were washed in serum-free media and fixed for 15 min in 4% paraformaldehyde in PBS. For microtubule staining, cells were fixed in −20°C methanol for 5 min. The following antibodies were used: rabbit anti-peptide antibody against APP C-terminus aa676–695 of APP695 (1∶2000, protein A purified IgG, 14 mg/ml Sigma A8717) or N-terminus aa46–60 of APP695 (1∶500, protein A purified IgG, 8.1 mg/ml, Sigma, A8967), amino acids); anti–APP-C aa673–695 of APP 695 (1∶1000, 0.25 mg/ml, affinity purified on the peptide, Zymed/Invitrogen 36–6900); mouse anti-VP5 antibody (IgG2b, 1∶1000, 3B6, EastCoast Bio); mouse anti-gE antibody (IgG2a at 1∶1000, East Coast Bio); mouse anti-gD antibody (IgG2a,1∶1000, East Coast Bio); mouse anti-LAMP2 (IgG1, 1∶200, Abcam); mouse anti-tubulin (IgG1, 1∶400, Sigma); sheep anti-TGN46 (1∶1000, AbD Serotec); rabbit anti-histone H3 (1∶200, protein A purified IgG, 0.9 mg/ml goat anti-rabbit IgG (Upstate Biologicals-Millipore); mouse anti-actin (1∶1000, Amersham/GE Healthcare) goat anti-rabbit Alexa-Fluor 555 (1∶2000, Molecular Probes); donkey anti-rabbit IgG Alexa-Fluor 555, donkey anti-sheep IgG Alexa-Fluor 647 and goat anti-mouse IgG Alexa-Fluor 647 (1∶2000, Molecular Probes); goat anti-mouse Cy5 (IgG2b, 1∶500, Jackson Labs); goat anti-mouse FITC (IgG2a, 1∶2000, Jackson Labs).

To minimize non-specific binding of antibodies from rabbit and sheep to viral Fc receptor, gE/gI [42], [98], we used a blocking buffer containing 20 µg/ml human IgG (Sigma), 3% BSA, 3% normal goat serum and 0.2% Triton X-100 in PBS (pH 7.4). For TGN46 staining, goat serum was omitted from the blocking buffer and the concentration of human IgG raised to 40 µg/ml. After blocking, cells were incubated for 1 hr in primary antibodies diluted in 3% BSA and 0.2% Triton X-100. After three washes in PBS, cells were incubated in secondary antibodies, washed in PBS and mounted in anti-quench with DAPI (0.001%) [99]. Non-specific staining by the secondary antibodies was monitored by omission of the primary antibodies in coverslips stained in parallel and then using the exposure times for stained samples that gave no signal in the secondary-only sample. Neither anti-gE nor anti-gD antibody stained mock-infected Vero cells. A Blast search (NCBI, NIH), found no HSV1 proteins with amino acid sequence homology to APP peptide antigens.

Fluorescence images were captured either by a 63X/1.4 N.A. oil immersion Plan Apochrome objective on a Zeiss Axioscope Z1 using the MRM AxioCam and AxioVision 4.5 software, or on a Zeiss LSM 410 confocal laser scanning microscope equipped with a krypton-argon laser for excitation at 488, 568, and 647 nm running Phoenix, v,2,0,2524 software. (Microcosm, Inc.). Confocal images of fixed cells used a pinhole adjusted to a narrow optical section (0.8 µm). Capture time was set by imaging coverslips stained in parallel with secondary only, and using linear grayscale for each channel. Figures were created using Photoshop CS2 and 3 (Adobe). For widefield deconvolution a z-stack was collected at 0.375 nm stepsize with 45 focal planes using th 63x objective. A region of interest was selected and deconvolved using AxioVision 4.5 DCI program using the automated PSF and interative processing. Deconvolved stacks were projected into 3D and rotated using AxioVision 4D Rendering software (Zeiss.com).

### Western blotting

After 9 hr, synchronously infected or mock-infected cell cultures (100 mm petri dish) were washed in warm serum-free media, and then scraped into lysis buffer (50 mM NaCl, 30 mM Tris-HCl (pH 7.2), 5 mM EDTA, 50 mM NaF, 2 mM sodium vandadate, 1 mM PMSF, 5 mM p-nitrophenylphosphate, 1% Nonidet-p-40), protein concentration measured by bicinchoninic acid kit (Sigma Aldritch), and volume adjusted to give equal protein concentrations across all samples which were then aliquoted into gel sample buffer and boiled for 5 min [79]. Parallel lanes of 10% polyacrylamide gels (BioRad) were loaded with equal total protein concentrations of HSV1 infected and mock-infected cell lysate and electrophoresed. Viral preparations were similarly loaded and run in 7.5% SDS gels. After overnight transfer in transfer buffer [17], blots were either stained for protein with amido black in methanol (Sigma) or blocked in Tris-buffered saline with 0.2% Tween and 5% instant milk, and probed for c-APP with anti-APP rabbit polyclonals (Zymed, Sigma, and Chemicon); for actin with anti-actin mouse monoclonal (Amersham) as a loading control; and for VP5, a 150 kDa abundant capsid protein (Virusys/Invitrogen) to detect the presence of virus. Banding patterns were detected by HRP-conjugated goat anti-rabbit-antibody or anti-mouse (Calbiochem) developed for enhanced chemiluminescence (Amersham/GE Healthcare) according to the manufacturer's instructions, with exposure to Kodak Xray films for 1, 5, 10, 20 min and over night. The position of the blot on the film was marked with black lab pen. Films were developed in a Kodak X-OMAT automated film processor. After developing, the film was placed on the nitrocellulose and the position of the pre-stained molecular weight markers indicated on the film. None of the antibodies used in this study detected new bands in HSV1 infected cells compared to uninfected cells. Detection of increased APP expression was variable, and best detected when protein concentrations were measured and equivalent amounts loaded from uninfected and infected cells.

### Quantitative analysis of co-localization

Quantitative co-localization analysis was performed on raw data using AxioVision 4.5 software in two ways: (1) Scatter plot, a graphical display that compares total pixel coincidence between two channels across the entire image. The intensities of two channels are distributed along the x- and y-axis in a scatter plot. If intensity of images in each channel completely overlaps, then the plot displays a straight diagonal lin,e starting from the origin of the scatter plot. This computational approach provides an average pixel-coincidence between two channels of the same field. (2) Linescan showsw a graph of the pixel intensity in each channel versus its position along a straight line drawn across a merged image. A superimposition of peaks between channels indicates high intensity overlap per pixel along the line. Linescans detect overlap along a line in a region of interest, while scatterplots can measure the global degree of co-incident intensities across a whole image field.

The number of fluorescent particles was counted in randomly selected images of synchronously infected cells by an independent person. All results are presented as mean ± standard error of the mean (SEM). Histograms were made from spreadsheets of counts using Microsoft Office Excel.

### Immunogold Electron-microscopy

Synchronously HSV1-infected Vero cell cultures were washed in warm serum-free media and fixed in 4% formaldehyde in 100 mM phosphate buffer overnight. Fixed cells were rinsed in PBS containing 0.15% glycine, and then scraped into PBS containing 5% bovine serum albumin using custom-made Teflon scrapers prepared from Teflon sheets. Scrapings were pelleted in an Eppendorf benchtop centrifuge, resuspended in warm PBS with 10% gelatin and 0.01% blue dextran (Sigma), re-pelleted, and cooled to 4°C to solidify the gelatin. The tips of the tubes containing a visibly blue pellet of cells were cut off, the cell pellets scooped out and post-fixed in buffered 4% formaldehyde for 30 min., cut into 0.1–2 mm cubes and soaked overnight in 2.3 M sucrose at 4°C. The next day, cell pellets were mounted onto metal specimen pins (Leica Microsystems Inc, Deerfield, IL), frozen in liquid nitrogen and placed in 100% methanol containing 1% uranyl acetate (SPI Inc) cooled to −80°C in an AFS2 Freeze Substitution Device (Leica Microsystems, Inc).

The AFS2 was programmed to warm to −60°C over 16 hr after which time the methanol/uranyl acetate was replaced with pre-cooled absolute ethanol. The specimen pins were removed from the solvent leaving the cell pellets to fall to the bottom of the tubes and the cell pellets were washed six times in fresh, cold ethanol over 6 hr. The cell pellets were then gradually warmed to −50°C and infiltrated with increasing amounts of Lowicryl HM20 resin (Electron Microscopy Sciences, Hatfield, PA) dissolved in ethanol and left overnight in a 1∶3 mixture of ethanol and resin. The next day, the cells were soaked in 3 changes of 100% Lowicryl resin and then placed in gelatin embedding capsules for polymerization. The cell pellets were covered with fresh 100% resin, labeled and polymerized under UV light at −50°C. After 5 days, the specimen blocks were gradually warmed to RT overnight.

Polymerized blocks, which were pink in color, were removed from the embedding capsules and placed overnight under UV illumination (in a laminar flow cabinet) at RT to allow unpolymerized volatile resin components to escape. Cells were sectioned with an Ultracut S ultramicrotome (Leica Microsystems Inc) equipped with a diamond knife (Diatome USA Inc). Sections were mounted on Formvar/carbon-coated metal specimen grids. After blocking with 20 µg/ml human IgG in PBS, sections were fixed with 1% gluteraldehdye for 10 min, quenched in 1% glycine in PBS for 10 min, blocked in PBS containing 10% BSA and 0.5% fish skin gelatin (blocker; Sigma), and then labeled with rabbit anti-cAPP antibodies (Sigma) or with irrelevant rabbit antibodies (rabbit anti-mouse IgG, Invitrogen) diluted 1∶20 in blocker, followed by 10 nm protein A-gold also diluted in blocker (University of Utrecht, The Netherlands). Omitting the human IgG block had no effect on the level of labeling. After immunogold staining, sections were contrasted with uranyl acetate and lead citrate [100]. The immunolabeled, contrasted sections were imaged using a Tecnai G2 20 transmission electron microscope operating at 80 kV. Images were collected using a 2 megapixel side-mounted digital camera (XR41; Advanced Microscopy Techniques) attached to the TEM.

### siRNA knock-down of APP

Three formulations of siRNA were purchased from Qiagen (Valencia, CA, USA): non-silencing siRNA sequence, shown by Basic Local Alignment Search Tool (BLAST) search not to share sequence homology with any known human mRNA; 2) specific siRNA against an unrelated gene product, MAPK-1, (Qiagen SI00300755); and specific siRNA against the APP target sequence CTGGTCTTCAATTACCAAGAA, an experimentally validated target (Qiagen, SI02780288). Knock-downs were performed according to the manufacturer's instructions in ARPE-19 cells and in Vero cells with minor modifications. Briefly subconfluent monolayers were grown in 12-well plates to a density of 1.2×10^5^ cells in 1100 µl of growth media per well. For each well, siRNA (15 ng in 100 ul optimen (Gibco/BRL/Invitrogen)) was mixed with 6 ul HiPerfect transfection reagent (Qiagen) and incubated for 10 min RT before being added to the cells. After removal of the media from the culture wells, the siRNA mixture was added (106 µl) to each well and the culture incubated overnight at 37°C. On day 2, the siRNA media was replaced with growth media and cultures incubated for another day to achieve optimal silencing by Western blotting. At 48 hr post-transfection, cultures were synchronously infected with VP26-GFP HSV1 or mock infected. At 7–9 hr p.i., cultures were either scraped into Western blot lysis buffer or fixed, stained for APP, gE or gD and DAPI, and imaged as described above.

### Live confocal imaging

Vero cells were grown on chambered glass coverslips (Lab-Tek, Nalge Nunc International) and transfected with the pMonoRed-APP695 plasmid, or with both pMonoRed-APP695 and pK26GFP for cells infected with the gEnull, gE wildtype (NS) or gE rescue virus, using 1 µg plasmid DNA/chamber containing 40–80,000 cells using lipofectamine2000/Optimem standard protocols (Invitrogen). After 48 hr incubation to allow for fusion protein expression, cells were synchronously infected with VP26-GFP HSV1 at 1–10 PFU/cell, and either fixed or imaged at 5–10 hr p.i.. Only cells with low levels of mRFP expression as determined by weak fluorescent signal, no aggregation of label in nuclear envelope, and clearly visible small, rapidly motile mRFP particles, as previously described for YFP-APP transfections [53], were selected for imaging. Because of variability between cells in a culture, quantification of expression levels on an individual cell basis would not be feasible. We did not test for normal glycosylation of the fusion protein since the APP-mRFP has almost identical distribution to the endogenous protein, and we therefore infer that it is normally processed.

Dynamic interactions between APP-mRFP particles and VP26-GFP-labeled capsids or viral particles were recorded under a 63X/1.4 N.A. oil objective, and time-lapse image sequences were collected at 3-second intervals simultaneously in FITC- and Cy3-fluorescent channels as well as a transmitted light channel, with 3 photo multiplier tubes using a Zeiss 410 confocal laser scanning microscope as described above. During observation, cells were maintained at 37°C through a temperature-controlled sample chamber and ring (Tempcontrol 37-2 digital, Carl Zeiss, Inc.). Movies from resulting time-lapse series were produced using NIH ImageJ (NIH).

Cells with low to moderate expression of APP-mRFP were selected for long-term imaging. Expression levels were determined by the intensity of the image after scanning at a fixed pinhole (40), gain (Detector gain: 80–120, Amplifier gain: 1–6) and Detector offset (3990). Our rationale was to avoid high levels of expression that could alter normal APP distribution. The large pinhole size gave an optical section of 12 µm that captured the full cell thickness. Most particled moved perpendicular to the optical section and remained within the filed of view. A few particles released to the apical (dorsal) cell surface remain visible until they separate from the cell and move out into the media.

### Transport analysis

Instantaneous velocity was measured from confocal sequences of synchronously infected cells using MetaMorph (Molecular Devices, Inc, Sunnyvale CA, USA). Videos of cells with active movements were selected for analysis. All mobile VP26-GFP and/or APP particles were measured inside each cell selected. Briefly, the distance moved between two consecutive frames was measured by marking the center of a fluorescent particle in each consecutive frame with a mouse-driven cursor. The distance in pixels was converted to real measurements in microns based on calibration (typically, 1 pixel represented 0.14 mm). To evaluate the accuracy of cursor marking and stage drift [101], we measured the movements of viral particles in fixed cells and of stationary viral particles in live cells from image sequences captured in time-lapse mode at the interval of 3 sec, as for our living moving particles. Mean displacements of GFP particles in fixed cells and stationary GFP particles in live cells were 0.18±0.09 mm (n = 890 measurements) and 0.27±0.09 mm (n = 990 measurements), respectively. Displacements of stationary GFP particles in live cells reflected our recording condition, and were thus used to set criteria defining pause versus movement. A normal distribution of the frame-to-frame displacements corresponding to 95% of the tracking error distribution (±2 SD) gave an interval score from 0.09 to 0.45 mm. Thus, any displacement of viral particles moving less than 0.45 mm (3 pixels) between consecutive frames was considered immeasurable. From this, only movements with instantaneous velocities greater than 0.15 mm/s were designated as runs and included in velocity calculations. Periods with instantaneous velocities between 0–0.15 mm/s for at least two consecutive frames were designated as pauses. Only particles moving into and out of a frame were counted.

## Supporting Information

Figure S1 **Quantitative validation of infection protocol.** (A) Distribution of viral particles in infected cells. Cells infected with VP26-HSV1, incubated for 7–9 hr p.i., fixed and stained for DAPI as in Figure 1. Numbers of cells in each of five categories representing distribution of viral particles as diagrammed were counted from digital images taken at random from 3 different experiments. After constitutive infection (96.2+/− 6.7% infected, top row) when virus remains in the medium throughout incubation, cells displayed GFP-labeled particles in all 5 distribution categories, including 29% that display >10 cytoplasmic particles with no evidence of nuclear GFP synthesis. These GFP particles thus must represent in-coming GFP-labeled virus. In parallel cultures infected for 1 hr with our protocol (87.5 +/− 24.4% infected, lower row), no cells were found with >10 cytoplasmic GFP-particles in the absence of nuclear GFP (boxed column), and a higher percentage of cells were found with only nuclear GFP, or both nuclear GFP and cytoplasmic GFP particles (lower row). Thus, after synchronized infection only small numbers of VP26-GFP particles are found in the cytoplasm of cells not expressing viral genes as evidenced by absence of viral-encoded VP26-GFP in the nucleus. (B) Average number of VP26-GFP labeled viral particles in the cytoplasm after synchronization. To determine the average number of incoming viral particles in the cytoplasm at later stages of infection, we counted particles at two time points (6.5–7 hr and 9 hr) after synchronized infection. At 6.5–7 hr p.i., the two predominant patterns in productively infected cells were: (1) few cytoplasmic VP26-GFP-particles whether or not there was nuclear GFP (3+/−3.0 and 3+/−2.3 cytoplasmic particles respectively); or (2) many cytoplasmic particles in cells with strong nuclear GFP (65+/−5 cytoplasmic particles). Less than 3% of cells displayed other distributions, and none had many cytoplasmic particles without nuclear GFP. At 9 hr p.i. all infected cells had strong nuclear GFP and many particles in the cytoplasm. Experiments were done in triplicate and counts (16,300 GFP particles) were made on at least three different coverslips for each condition for no less than 10 microscopic fields per coverslip.
(TIF)

Figure S2 **The majority of VP26-GFP cytoplasmic particles represent viral capsids.** (A) Co-localization of VP26-GFP particles with the VP5 capsid protein in the cytoplasm. Cells infected with VP26-GFP HSV1 (green) at 7–9 hr p.i. were fixed and immuno-stained for VP5 (red). Most cytoplasmic particles appear yellow as they are labeled with both fluorochromes. (B and C) High magnification of the boxed region in (A) showing the individual channels of VP26-GFP (B, green) and anti-VP5 (C, red). Of 27 GFP particles, 24 also stain for VP5 in this region. Arrowheads indicate the few GFP-particles not stained for VP5. Since VP26 coats the outside of particles after capsidation, some particles will be expected to stain for VP5 but not yet acquired VP26-GFP. Conversely, VP26 may mask VP5 antibody-binding sites. Such particles do not stain consistently with anti-VP5 antibody [31]. For this high level of combined staining, we modified the fixative to include the detergent 0.2% Triton. This improved VP5-capsid antibody staining, further suggesting that viral capsids are inside a detergent-soluble membrane compartment. (D) A linescan showing coincidence of the peaks of pixel intensity of VP26-GFP and VP5 channels. (E) Histogram of the percentage of VP26-GFP particles co-localized with VP5. Note 92.8±2.9% of VP26-GFP cytoplasmic particles (n = 2927) co-localize with VP5.
(TIF)

Figure S3 **Split channels of** **Figure 2** **A and B.**
(TIF)

Figure S4 **Successful blocking of non-specific binding of antibodies to the HSV1 Fc receptor, gE.** (A) Cells synchronously infected with VP26-GFP HSV1 (green) were fixed and stained with rabbit antibody against histone H3, purified the same way and diluted to the same concentration as the Sigma rabbit anti-APP used in this paper. Cells were routinely stained in parallel for all figures presented here for histone and for APP, with identical blocking, incubations, washes, and secondary antibodies. Images were captured with the same exposure settings. Note that VP26-GFP viral particles in the cytoplasm are not stained with the histone antibody while the nucleus is appropriately stained. Thus the secondary antibody has no anti-viral activity, and the blocker successfully eliminates Fc binding by the antibodies. (B) Histogram showing a quantitative analysis of the immunostaining of anti-histone antibodies (red). Most (86.9±7.2%) viral particles (green) are not stained for histone (red). We counted 4791 viral particles in 19 cells from 3 independent experiments.
(TIF)

Figure S5 **Co-localization of viral capsids (VP26-GFP, green), viral envelope (gD, blue) and APP (red) after synchronous infection with VP26-GFP HSV1.** This figure is in parallel to Figure 4, showing results for viral glycoprotein gD similar to those obtained for the other viral envelope glycoprotein, gE, at the same time point. As for gE, the majority of the VP26-GFP particles stained for both gD and APP. (A) An example of infected cells stained for gD (blue) and APP (red). (B) High magnification of the boxed regions in (A). Arrows indicate those particles with all three labels. Arrowheads indicate only gD (blue) or APP (pink). (C) Intensity profile along a line (white) drawn across the merged image in (A). Arrows indicate the superposition of peaks for each channel. (D) Histograms showing the percentage of VP26-GFP particles in each category. VP26-GFP alone (4.9±3.2%), with APP (4.0±2.4%), with gD (22.2±5.9%), and with both APP and gD (69.3±7.6%). Experiments were performed in triplicate, and 2228 particles in 10 cells were counted from each experiment were counted.
(TIF)

Figure S6 **Gallery of viral configurations by thin section immuno-gold electronmicroscopy showing abundant gold particles decorating intracellular viral particles.** (A and C) Examples of APP-gold labeling of various types of viral particle-membrane configurations, including clusters that were also surrounded by an APP-gold labeled membrane. (B and D) Non-relevant polyclonal rabbit antibodies did not label these clusters or their surrounding membrane, nor other configurations of virus and cellular membrane systems. (E) Gallery of examples of APP-gold decorated viral particles. Scale bars = 100 nm.
(TIF)

Figure S7 **Cytoplasmic viral particles are associated with microtubules.** Cells mock-infected or infected with VP26-GFP HSV1 (10 pfu/cell) at 7–9 hr p.i. were stained for β-tubulin (red) and the nucleus with DAPI (blue). Images were captured with confocal microscopy. (A) Normal microtubule distribution in mock-infected cells. Microtubule organizing centers (MTOC) were clearly seen at one side of the nucleus. (B) Abnormal microtubule distribution in HSV1-PV26-GFP infected cells at 7–9 hr p.i. Note that all cells with VP26-GFP display similar microtubule disarray. (C) A high magnification zoom of a representative infected cell showing many VP26-GFP particles in the cytoplasm apparently associated with microtubules (arrowheads) (87±0.1%, n = 136 particles from 4 cells).
(TIF)

Figure S8 **Out-going HSV1 particles display a wide range of behavior.** A) A gallery of movements of HSV1-APP double-labeled particles (arrow) from another infected cell similar to one the shown in Figure 8. Time-lapse sequences were captured at 3-sec intervals. The last panel shows the trajectory of the HSV1-APP vesicle. See **Movie S5**. (B) Another gallery of movements of HSV1-APP tubules (arrow). Shown is a VP26-GFP particle (green) moving within or upon a large APP-mRFP (red) tubule. The APP tubule changes its shape during the sequence. This is a region of interest taken from the infected cell shown in **Figure 8a-b** and Movie S2.
(TIF)

Table S1 Comparison of movements of VP26-GFP and APP-mRFP singly and together*.
(PDF)

Movie S1 **APP stains gEnull viral particles.** This sequence shows the 3D rendering of a z-stack of the cell shown in Figure 3 that was infected with gE null virus (green) and stained for APP (red) and DAPI (blue).
(MOV)

Movie S2 **Transport of VP26-GFP particles with APP-mRFP vesicles during viral egress.** The cell expressing APP-mRFP was infected with VP26-GFP HSV1 at 7 hr p.i.. The movie is from a 300-frame sequence, taken at 3-sec intervals, and displayed at 9 frames per second. A still frame and track tracing from this video are shown in **Figure 8a and b**.
(MOV)

Movie S3 **An example of VP26-GFP particles transporting with APP-mRFP** selected from Movie S1 showing the movement of a single VP26-GFP-APP-mRFP (particle 1, as indicated in Fig. 8b). The VP26-GFP/APPmRFP double-label particle moves away from the nucleus towards the plasma membrane at variable rates ranging from ∼0.22–1.03 µm/sec with no pauses. See **Figure 9a** for a gallery of these movements.
(MOV)

Movie S4 **Another example of VP26-GFP particles transporting with APP-mRFP** selected from Movie S1. The movement of a single VP26-GFP-APP-mRFP (particle number 2 as indicated in Figure 8b). The VP26-GFP/APPmRFP double-label particle moves away from the nucleus towards the plasma membrane at highly variable rates with many pauses, again ranging from ∼0.22–1.03 µm/sec. See **Figure 9b** for a gallery of these movements.
(MOV)

Movie S5 **Transport of VP26-GFP particles with APP-mRFP captured from another experiment.** Arrows show the movements of VP26-APP double-label particles. Movie sequences were captured at 7–9 hr p.i. with 3-sec time-lapse intervals for 219 frames. See **Figure S8A** for a gallery of still frames of these movements.
(MOV)

Movie S6 **APP-mRFP associates with gEnull virus labeled with VP26-GFP.** Cells were first dually transfected with pAPP-mRFP and pK26GFP, infected 48 hr later with gEnull virus and then maged at 7–10 hr p.i.. Shown is a 51-frame time-lapse sequence captured at 3-sec intervals of two adjacent cells expressing both labels and infected with the gEnull virus.
(MOV)
