# Supplementary figures and images for: Herpes Simplex Virus Dances with Amyloid Precursor Protein while Exiting the Cell
Source: PLoS One. 2011 Mar 31;6(3):e17966. doi: 10.1371/journal.pone.0017966 (PMC3069030; doi:10.1371/journal.pone.0017966)

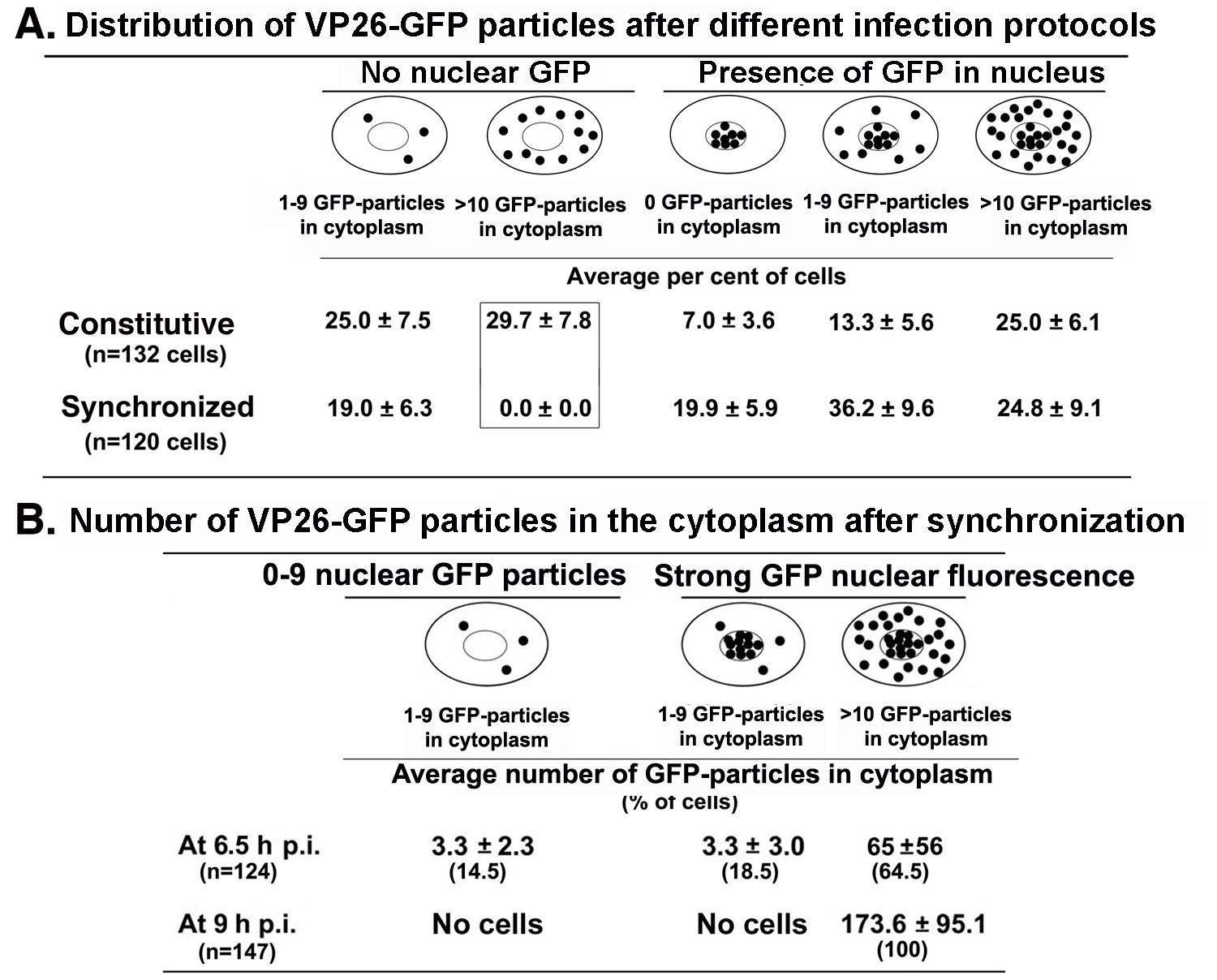

Supplement: Figure S1 — Quantitative validation of infection protocol. (A) Distribution of viral particles in infected cells. Cells infected with VP26-HSV1, incubated for 7–9 hr p.i., fixed and stained for DAPI as in Figure 1. Numbers of cells in each of five categories representing distribution of viral particles as diagrammed were counted from digital images taken at random from 3 different experiments. After constitutive infection (96.2+/− 6.7% infected, top row) when virus remains in the medium throughout incubation, cells displayed GFP-labeled particles in all 5 distribution categories, including 29% that display >10 cytoplasmic particles with no evidence of nuclear GFP synthesis. These GFP particles thus must represent in-coming GFP-labeled virus. In parallel cultures infected for 1 hr with our protocol (87.5 +/− 24.4% infected, lower row), no cells were found with >10 cytoplasmic GFP-particles in the absence of nuclear GFP (boxed column), and a higher percentage of cells were found with only nuclear GFP, or both nuclear GFP and cytoplasmic GFP particles (lower row). Thus, after synchronized infection only small numbers of VP26-GFP particles are found in the cytoplasm of cells not expressing viral genes as evidenced by absence of viral-encoded VP26-GFP in the nucleus. (B) Average number of VP26-GFP labeled viral particles in the cytoplasm after synchronization. To determine the average number of incoming viral particles in the cytoplasm at later stages of infection, we counted particles at two time points (6.5–7 hr and 9 hr) after synchronized infection. At 6.5–7 hr p.i., the two predominant patterns in productively infected cells were: (1) few cytoplasmic VP26-GFP-particles whether or not there was nuclear GFP (3+/−3.0 and 3+/−2.3 cytoplasmic particles respectively); or (2) many cytoplasmic particles in cells with strong nuclear GFP (65+/−5 cytoplasmic particles). Less than 3% of cells displayed other distributions, and none had many cytoplasmic particles without nuclear GFP. At 9 h [file pone.0017966.s001.tif]

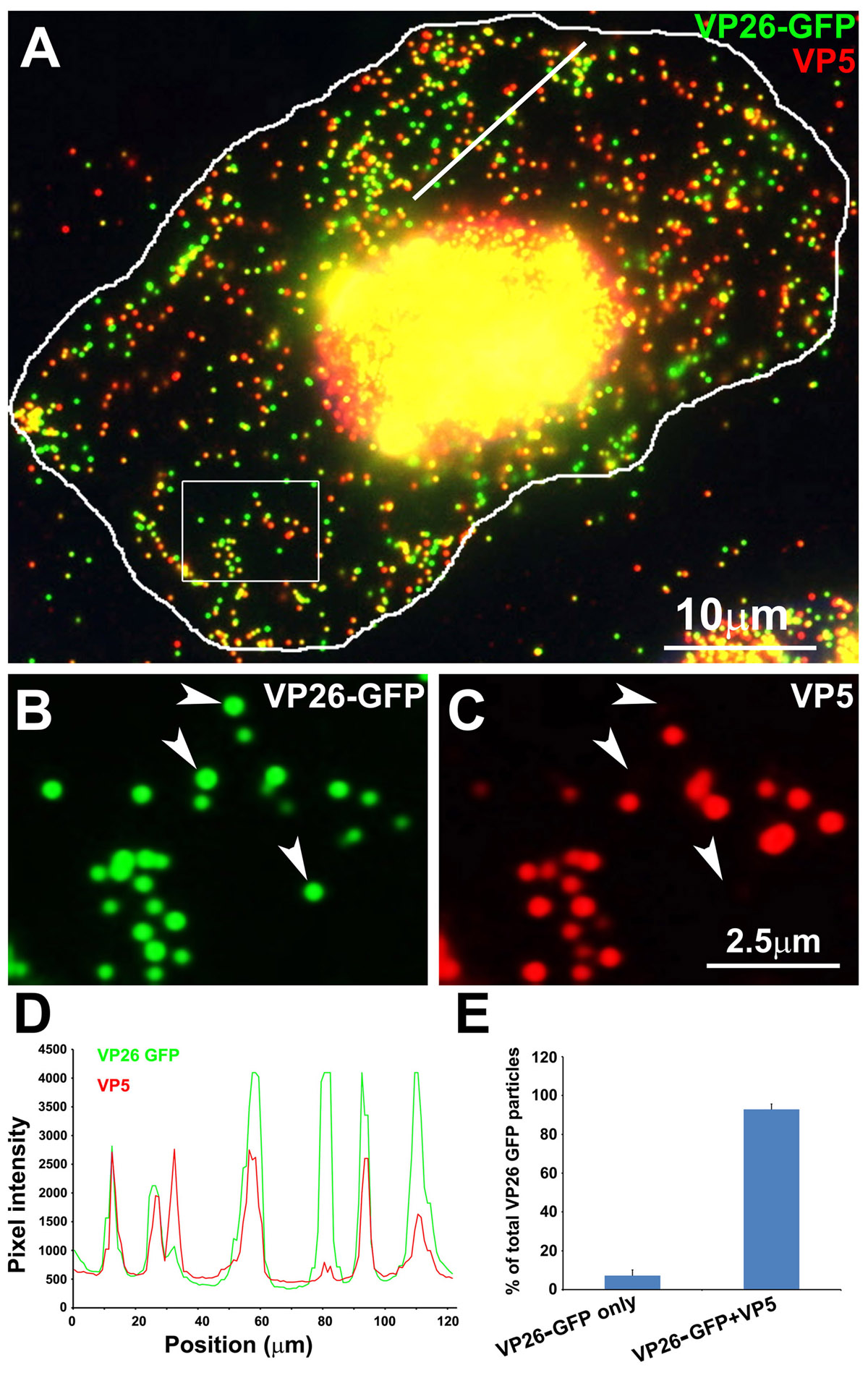

Supplement: Figure S2 — The majority of VP26-GFP cytoplasmic particles represent viral capsids. (A) Co-localization of VP26-GFP particles with the VP5 capsid protein in the cytoplasm. Cells infected with VP26-GFP HSV1 (green) at 7–9 hr p.i. were fixed and immuno-stained for VP5 (red). Most cytoplasmic particles appear yellow as they are labeled with both fluorochromes. (B and C) High magnification of the boxed region in (A) showing the individual channels of VP26-GFP (B, green) and anti-VP5 (C, red). Of 27 GFP particles, 24 also stain for VP5 in this region. Arrowheads indicate the few GFP-particles not stained for VP5. Since VP26 coats the outside of particles after capsidation, some particles will be expected to stain for VP5 but not yet acquired VP26-GFP. Conversely, VP26 may mask VP5 antibody-binding sites. Such particles do not stain consistently with anti-VP5 antibody [31]. For this high level of combined staining, we modified the fixative to include the detergent 0.2% Triton. This improved VP5-capsid antibody staining, further suggesting that viral capsids are inside a detergent-soluble membrane compartment. (D) A linescan showing coincidence of the peaks of pixel intensity of VP26-GFP and VP5 channels. (E) Histogram of the percentage of VP26-GFP particles co-localized with VP5. Note 92.8±2.9% of VP26-GFP cytoplasmic particles (n = 2927) co-localize with VP5. (TIF) [file pone.0017966.s002.tif]

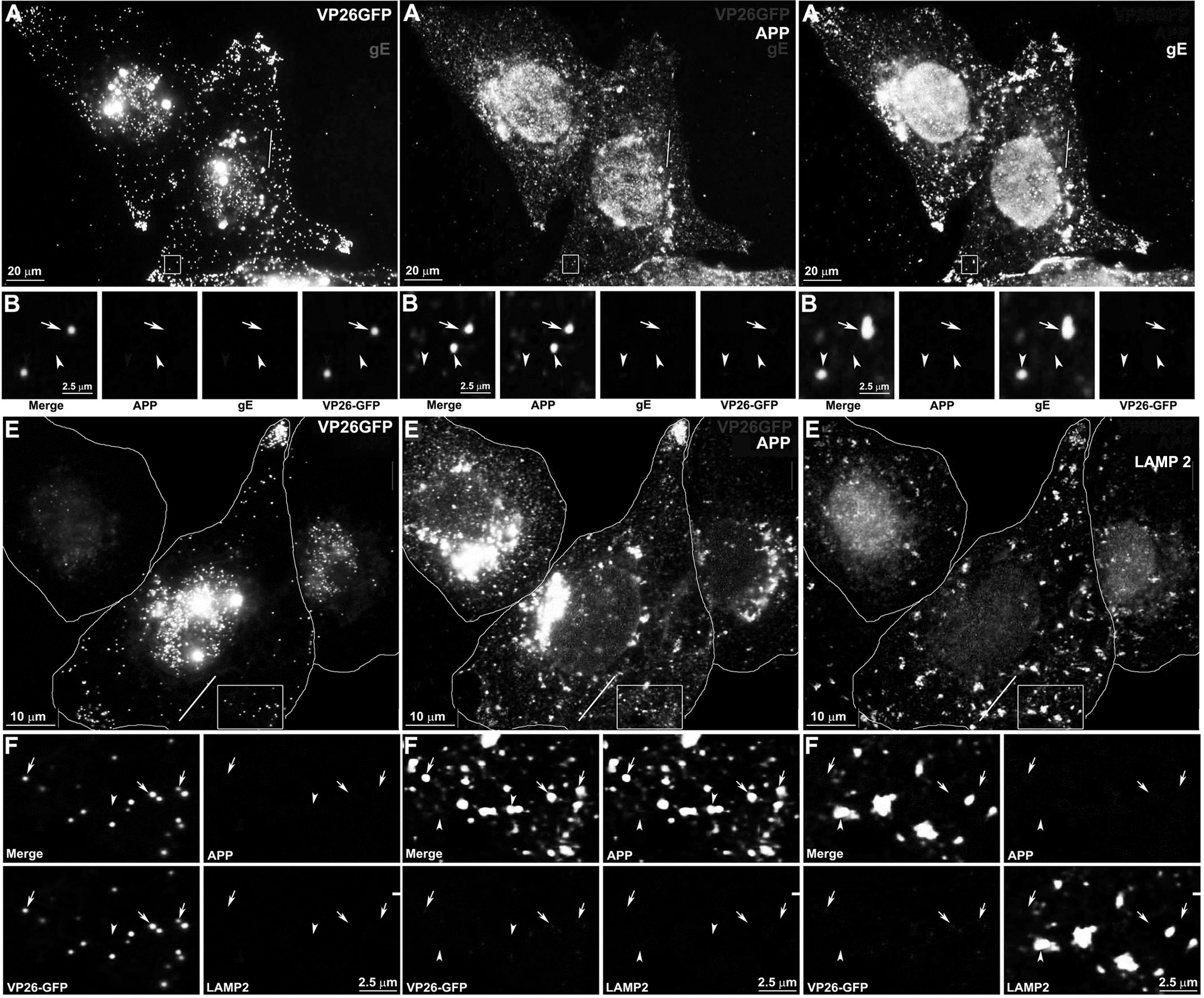

Supplement: Figure S3 — Split channels of Figure 2 A and B. (TIF) [file pone.0017966.s003.tif]

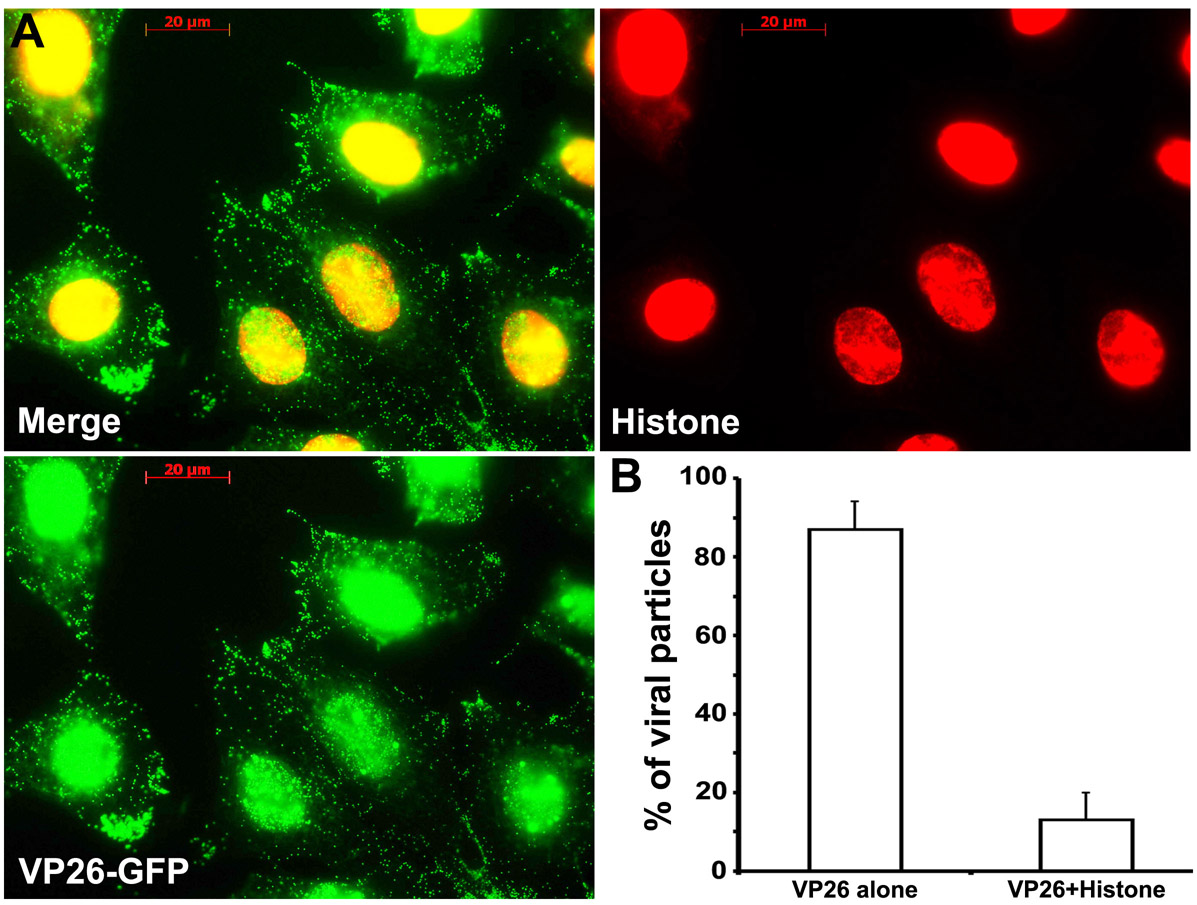

Supplement: Figure S4 — Successful blocking of non-specific binding of antibodies to the HSV1 Fc receptor, gE. (A) Cells synchronously infected with VP26-GFP HSV1 (green) were fixed and stained with rabbit antibody against histone H3, purified the same way and diluted to the same concentration as the Sigma rabbit anti-APP used in this paper. Cells were routinely stained in parallel for all figures presented here for histone and for APP, with identical blocking, incubations, washes, and secondary antibodies. Images were captured with the same exposure settings. Note that VP26-GFP viral particles in the cytoplasm are not stained with the histone antibody while the nucleus is appropriately stained. Thus the secondary antibody has no anti-viral activity, and the blocker successfully eliminates Fc binding by the antibodies. (B) Histogram showing a quantitative analysis of the immunostaining of anti-histone antibodies (red). Most (86.9±7.2%) viral particles (green) are not stained for histone (red). We counted 4791 viral particles in 19 cells from 3 independent experiments. (TIF) [file pone.0017966.s004.tif]

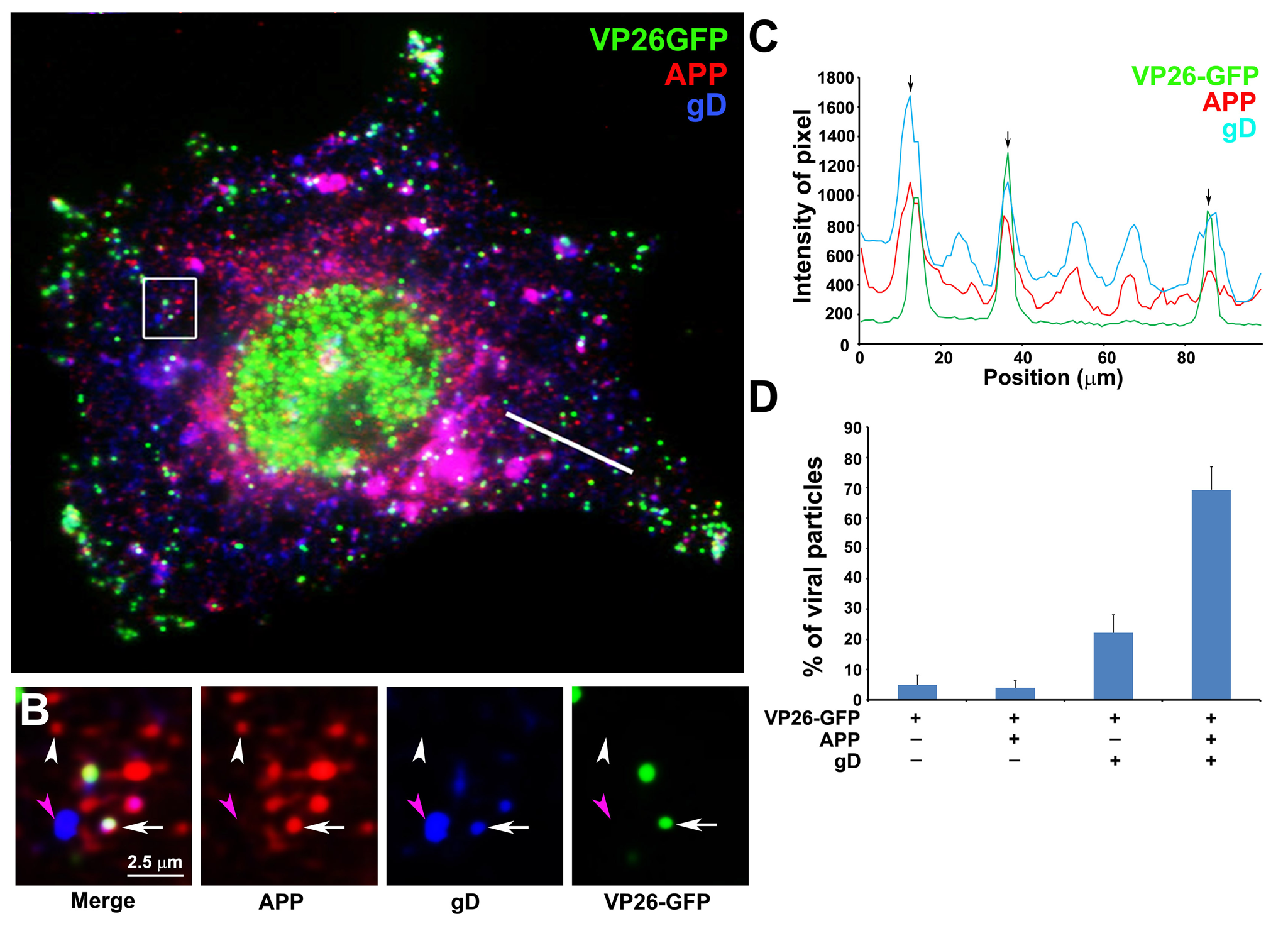

Supplement: Figure S5 — Co-localization of viral capsids (VP26-GFP, green), viral envelope (gD, blue) and APP (red) after synchronous infection with VP26-GFP HSV1. This figure is in parallel to Figure 4, showing results for viral glycoprotein gD similar to those obtained for the other viral envelope glycoprotein, gE, at the same time point. As for gE, the majority of the VP26-GFP particles stained for both gD and APP. (A) An example of infected cells stained for gD (blue) and APP (red). (B) High magnification of the boxed regions in (A). Arrows indicate those particles with all three labels. Arrowheads indicate only gD (blue) or APP (pink). (C) Intensity profile along a line (white) drawn across the merged image in (A). Arrows indicate the superposition of peaks for each channel. (D) Histograms showing the percentage of VP26-GFP particles in each category. VP26-GFP alone (4.9±3.2%), with APP (4.0±2.4%), with gD (22.2±5.9%), and with both APP and gD (69.3±7.6%). Experiments were performed in triplicate, and 2228 particles in 10 cells were counted from each experiment were counted. (TIF) [file pone.0017966.s005.tif]

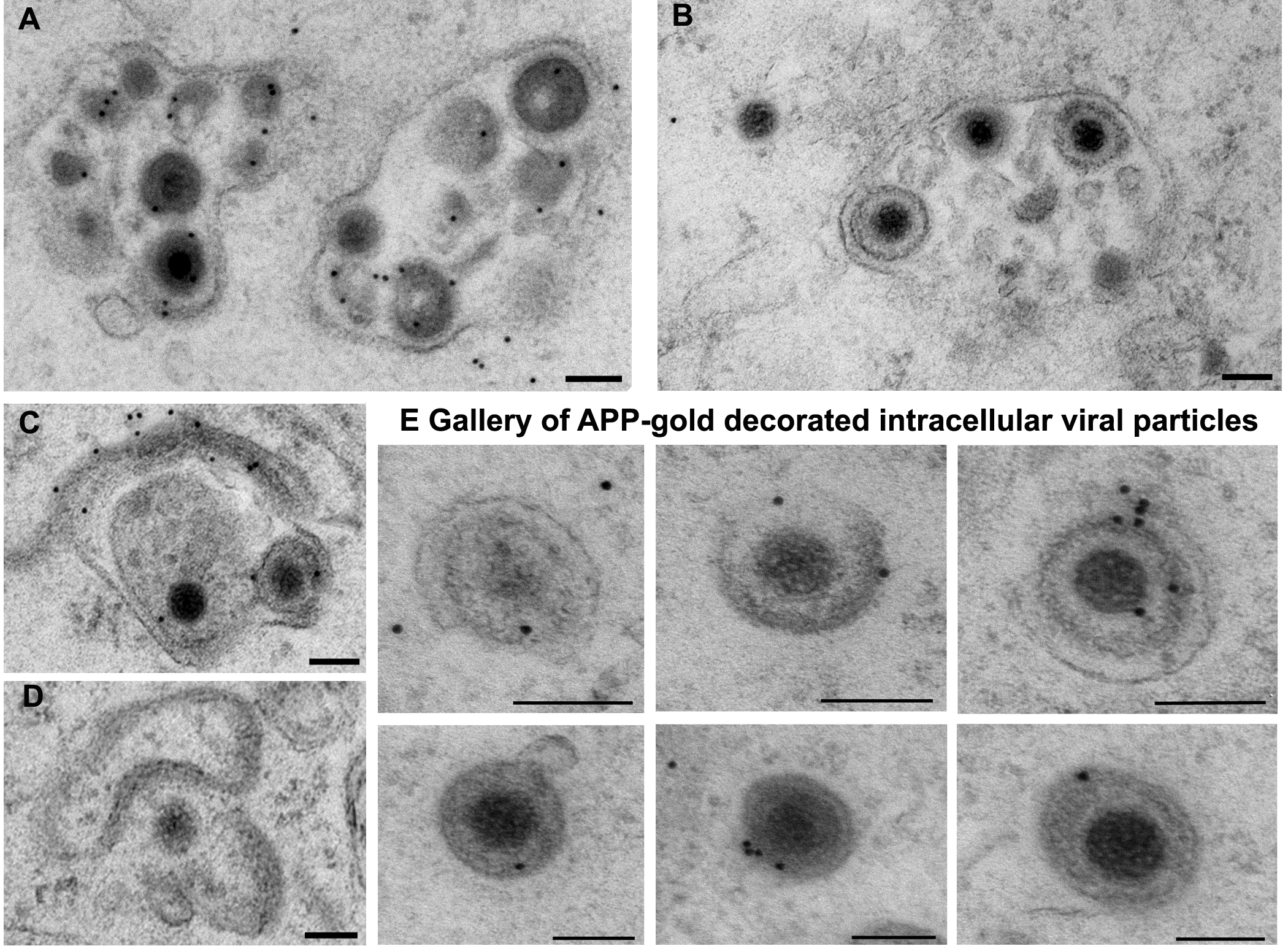

Supplement: Figure S6 — Gallery of viral configurations by thin section immuno-gold electronmicroscopy showing abundant gold particles decorating intracellular viral particles. (A and C) Examples of APP-gold labeling of various types of viral particle-membrane configurations, including clusters that were also surrounded by an APP-gold labeled membrane. (B and D) Non-relevant polyclonal rabbit antibodies did not label these clusters or their surrounding membrane, nor other configurations of virus and cellular membrane systems. (E) Gallery of examples of APP-gold decorated viral particles. Scale bars = 100 nm. (TIF) [file pone.0017966.s006.tif]

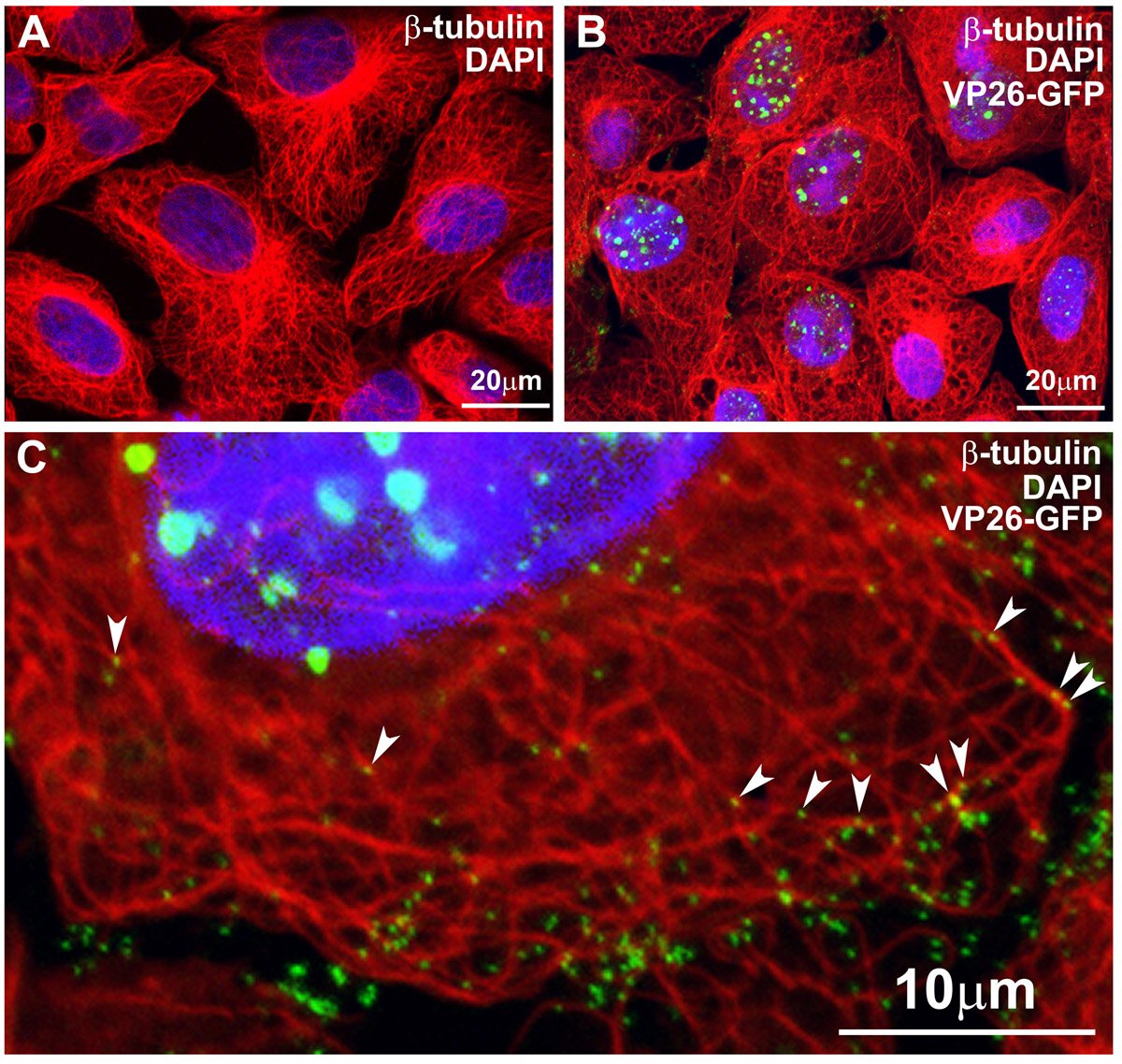

Supplement: Figure S7 — Cytoplasmic viral particles are associated with microtubules. Cells mock-infected or infected with VP26-GFP HSV1 (10 pfu/cell) at 7–9 hr p.i. were stained for β-tubulin (red) and the nucleus with DAPI (blue). Images were captured with confocal microscopy. (A) Normal microtubule distribution in mock-infected cells. Microtubule organizing centers (MTOC) were clearly seen at one side of the nucleus. (B) Abnormal microtubule distribution in HSV1-PV26-GFP infected cells at 7–9 hr p.i. Note that all cells with VP26-GFP display similar microtubule disarray. (C) A high magnification zoom of a representative infected cell showing many VP26-GFP particles in the cytoplasm apparently associated with microtubules (arrowheads) (87±0.1%, n = 136 particles from 4 cells). (TIF) [file pone.0017966.s007.tif]

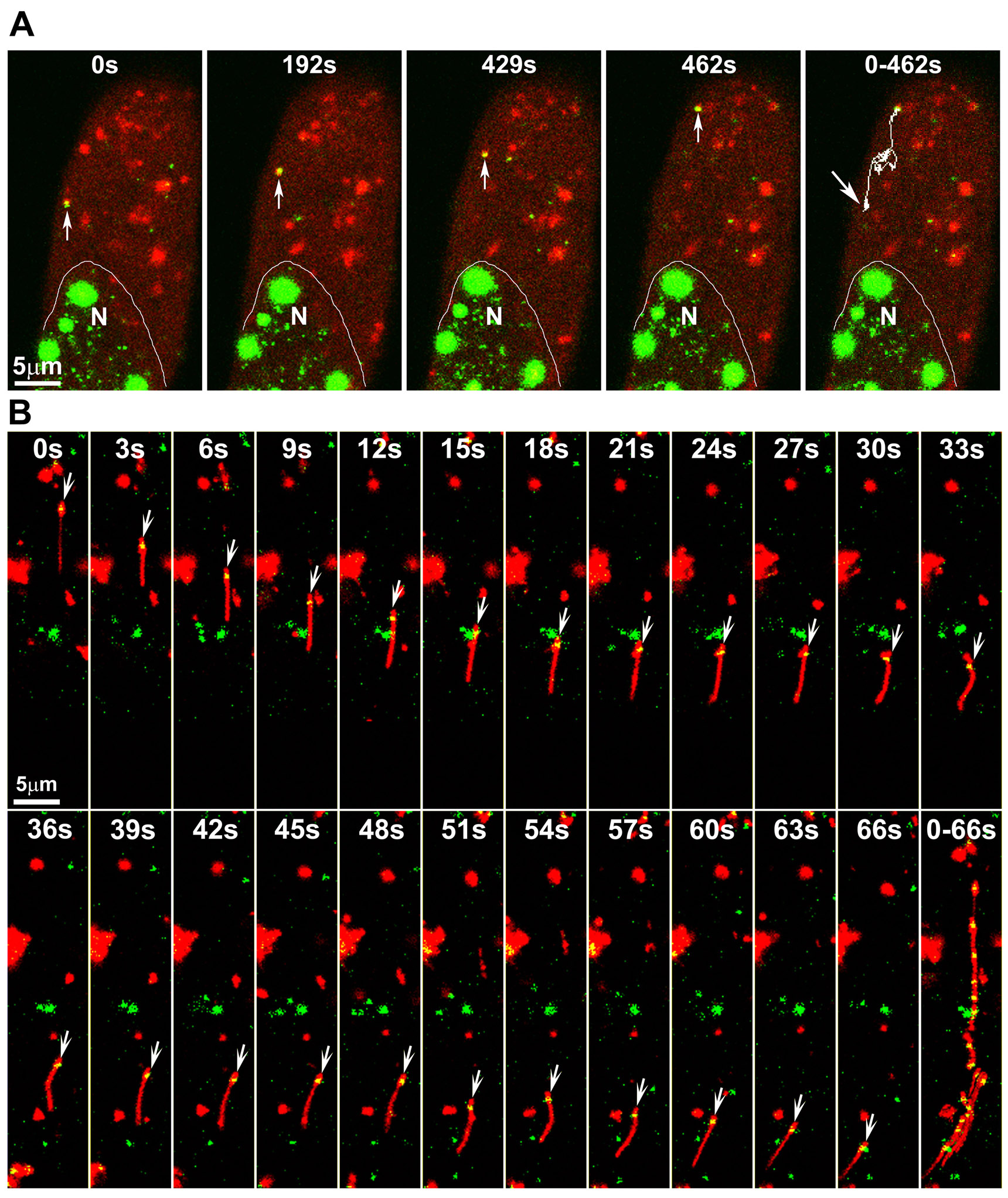

Supplement: Figure S8 — Out-going HSV1 particles display a wide range of behavior. A) A gallery of movements of HSV1-APP double-labeled particles (arrow) from another infected cell similar to one the shown in Figure 8. Time-lapse sequences were captured at 3-sec intervals. The last panel shows the trajectory of the HSV1-APP vesicle. See Movie S5. (B) Another gallery of movements of HSV1-APP tubules (arrow). Shown is a VP26-GFP particle (green) moving within or upon a large APP-mRFP (red) tubule. The APP tubule changes its shape during the sequence. This is a region of interest taken from the infected cell shown in Figure 8a-b and Movie S2. (TIF) [file pone.0017966.s008.tif]
