# Supplementary material for: Herpes Simplex Virus Dances with Amyloid Precursor Protein while Exiting the Cell
Source: PLoS One. 2011 Mar 31;6(3):e17966. doi: 10.1371/journal.pone.0017966 (PMC3069030; doi:10.1371/journal.pone.0017966)
Supplement: Table S1 — Comparison of movements of VP26-GFP and APP-mRFP singly and together*. (PDF) [file pone.0017966.s009.pdf]

SUPPORTING TABLE

Table S1. Comparison of movements of VP26-GFP and APP-mRFP singly and together*

	Average instantaneous velocity (AIV) (μm/s)	Range of AIV (μm/s)	Maximal velocity (μm/s)	Pauses		Run length (μm)
				% Total time	Duration (S)	
**Infected cells**						
VP26 and APP (n=118)	0.4±0.1	0.3-1.0	2.0	30.2±12.0	3-24	0.5-30.1
VP26 alone (n=15)	0.3±0.1	0.2-0.3	1.2	25.9±8.3	3-6	0.5-11.5
APP alone (n=64)	0.3±0.1	0.2-0.4	1.3	22.5±7.5	3-39	0.5-23.7
**Uninfected cells**						
APP alone (n=23)	1.1±0.2	0.3-1.3	4.2	4.4±1.2	3-12	13.2-149.7

* Movements of VP26GFP-HSV particles alone or with APP-mRFP and of APP-mRFP vesicles alone inside infected or uninfected cells were measured using MetaMorph from at least 3 different cells in independent movies. Particles moving through at least 3 frames are designated as moving particles. Only particles moving into and out of a frame were measured.
